# Ubc13: the Lys63 ubiquitin chain building machine

**DOI:** 10.18632/oncotarget.10948

**Published:** 2016-07-29

**Authors:** Curtis D. Hodge, Leo Spyracopoulos, J. N. Mark Glover

**Affiliations:** ^1^ Department of Biochemistry, University of Alberta, Edmonton, Alberta, Canada

**Keywords:** Ubc13, ubiquitination, Mms2, Uev1A, RING E3 ligase

## Abstract

Ubc13 is an ubiquitin E2 conjugating enzyme that participates with many different E3 ligases to form lysine 63-linked (Lys63) ubiquitin chains that are critical to signaling in inflammatory and DNA damage response pathways. Recent studies have suggested Ubc13 as a potential therapeutic target for intervention in various human diseases including several different cancers, alleviation of anti-cancer drug resistance, chronic inflammation, and viral infections. Understanding a potential therapeutic target from different angles is important to assess its usefulness and potential pitfalls. Here we present a global review of Ubc13 from its structure, function, and cellular activities, to its natural and chemical inhibition. The aim of this article is to review the literature that directly implicates Ubc13 in a biological function, and to integrate structural and mechanistic insights into the larger role of this critical E2 enzyme. We discuss observations of multiple Ubc13 structures that suggest a novel mechanism for activation of Ubc13 that involves conformational change of the active site loop.

## INTRODUCTION

Ubiquitination is a prime example of how evolution has exploited the use of small proteins as signaling molecules. The larger size of a protein compared with signaling modifications such as phosphorylation, methylation or acetylation provides more complexity and consequentially a wider range of utility than the former small modifications. Of course there are benefits to small and large post-translational modifications (PTMs), hence the existence of both. Ubiquitin is a ~8 kDa protein that can be conjugated to other proteins by its C-terminal carboxylate through the formation of an isopeptide bond resulting in a monoubiquitinated substrate [1, 2]. It can also be used to form homogenous, mixed, linear or branched polyubiquitin chains through successive isopeptide bond formation using one of its seven lysine residues (Lys6, Lys11, Lys27, Lys29, Lys33, Lys48, Lys63) or its N-terminal methionine (Met1) [1, 2].

There is a generally accepted E1-E2-E3 enzymatic cascade that is used to create most ubiquitin chains, with linkage specificity based on the particular E2 conjugating enzyme (E2) or homology to E6AP C terminus (HECT) E3 used [3–7]. The cascade commences with an E1 activating enzyme (E1) that catalyzes formation of an AMP-ubiquitin covalent intermediate, which activates the ubiquitin C-terminal carboxylate for subsequent transfer to the E1. The sulfhydryl of the E1 active site cysteine attacks the ubiquitin adenylate, forming a covalent thioester linkage to the ubiquitin molecule (E1~Ub). The next enzyme in the pathway, the E2 or ubiquitin conjugating enzyme, binds the E1 and the ubiquitin is transferred to the E2 active site cysteine in a trans-thioesterification reaction to form an E2~Ub conjugate. The last step in the cascade is achieved using an E3 ubiquitin ligase (E3). The E3 is responsible for providing target specificity by bringing the E2~Ub into close proximity with the target protein so that the ubiquitin can finally be transferred to a target lysine residue through the formation of an isopeptide linkage between the ε-amino group of the lysine and the C-terminal ubiquitin carboxylate. In the case of HECT E3s, the ubiquitin is transferred from the active site cysteine of the E2 enzyme to an active site cysteine residue in the HECT E3, which is subsequently transferred to a target lysine residue [7–9]. Polyubiquitin chains are formed by repeating the E1-E2 step with stimulation by a really interesting new gene (RING) E3 or E1-E2-E3 steps with a HECT E3, where the donor ubiquitin C-terminus is linked to an acceptor ubiquitin lysine instead of the lysine residue of the substrate (target) protein [3–6]. General E2 conjugating enzyme mechanisms, regulation, and biology have been reviewed [10, 11].

## MECHANISM OF LYS63-LINKED UBIQUITIN CHAIN SYNTHESIS

**Figure 1:**
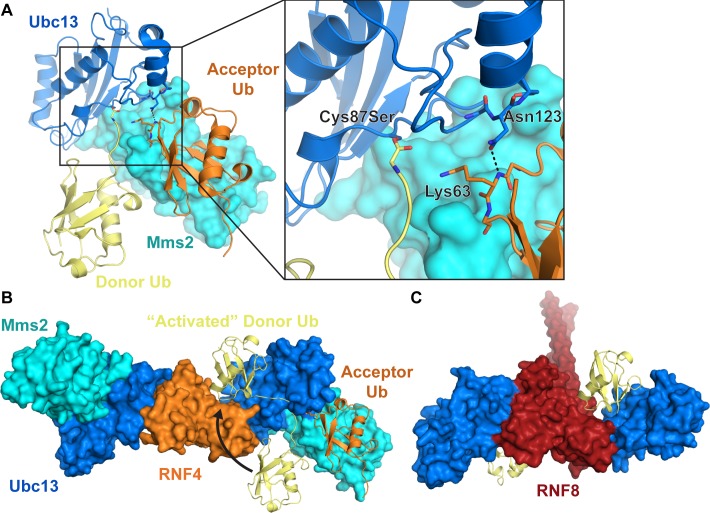
Formation of Lys63-linked ubiquitin chains by Ubc13/Mms2 and a RING E3 ligase **A.** Nucleophilic attack by the Mms2-bound acceptor ubiquitin Lys63 on the E2-ubiquitin thioester forms a Lys63-linked diubiquitin chain. RING E3s such as **B.** RNF4 (PDB: 5AIT with non-activated and acceptor ubiquitin from 2GMI) and **C.** RNF8 (PDB: 4WHV) bind to Ubc13 and bias the donor ubiquitin conformational distribution, which likely optimizes the geometry of the thioester relative to the incoming lysine. Ubc13 is blue, Mms2 is cyan, donor ubiquitin is yellow, acceptor ubiquitin is orange. Residue numbers in Ubc13/Mms2 correspond to human Ubc13 on the yeast structure 2GMI in **A.**

The chemical formation of a Lys63-linked ubiquitin chain is achieved by the E2 Ubc13 together with either of two non-catalytic, E2-like partner proteins, Mms2, which participates in nuclear Lys63-linked ubiquitin chain formation, or Uev1A, which is nearly identical to Mms2 and is involved in cytoplasmic ubiquitination [12, 13]. Lys63-linked ubiquitin chains can also be formed by the HECT E3 ligases yeast Rsp5 [14, 15] and human Nedd4-1 [16] and Itch/AIP4 [17], regardless of the E2 enzyme used [18]. A number of studies have revealed in structural detail how Ubc13 participates with Mms2 or Uev1A to build Lys63-linked polyubiquitin (Figure 1A) [19–23]. The previously mentioned E1-E2 trans-thioesterification reaction results in a donor ubiquitin covalently linked to the active site cysteine (Cys87) of Ubc13. A second acceptor ubiquitin molecule binds to Mms2/Uev1A non-covalently, which positions the ubiquitin Lys63 for attack on the Ubc13^C87^-donor ubiquitin thioester linkage [19, 24–26]. The acceptor ubiquitin Lys63 is directly engaged by Ubc13 Asn123, which may drive a conformational change in the Ubc13 active site loop to accommodate the incoming Lys63 (Figure 1A) [27]. The Mms2-ubiquitin non-covalent interaction is largely mediated by the canonical hydrophobic patch on ubiquitin (Leu8, Ile44, Val70) and a surface on Mms2 composed of residues Met54, Ile56, and Ile67 [19, 28, 29]. Suppression of the pKa and deprotonation of the ubiquitin Lys63 promotes nucleophilic attack on the thioester resulting in the formation of an isopeptide bond [30]. This reaction likely forms an oxyanion thioester intermediate, and the developing negative charge on the carbonyl oxygen is thought to be stabilized by the conserved Ubc13 Asn79 [31].

Given the mechanism described above, it is unlikely that the Ubc13/E2-variant heterodimer directly monoubiquitinates substrate lysine residues. It is more likely that Ubc13 extends existing chains or builds them on previously monoubiquitinated substrates. Indeed, Soss et al. [32] showed that in contrast to other E2 enzymes, Ubc13/Uev1A alone could not modify the E3 ligase CHIP *in vitro*, but created free ubiquitin chains. If, however, the E2 enzymes Ube2E1 or Ube2W that monoubiquitinate CHIP and Hsp70, were included in the reactions to prime the substrates with monoubiquitin, the substrates were polyubiquitinated. The apparent inability of Ubc13/E2-variant to directly ubiquitinate substrates is further highlighted by Mattiroli et al. [33], which demonstrated that Ubc13/Mms2/RNF8 efficiently extends monoubiquitinated substrate histone H2A *in vitro*, but not unmodified H2A. It is likely then, that other E2 enzymes first prime substrate lysine residues in cellular studies that implicate Ubc13 in modifying proteins with Lys63-linked polyubiquitin chains. The possibility exists that if the heterodimer of Ubc13 with Mms2/Uev1A is disrupted that Ubc13 could be used to directly monoubiquitinate substrates, however in this unlikely case the Lys63-linked polyubiquitination ability would also be abolished.

Ubc13 is likely the only E2 enzyme that requires the presence of a non-catalytic E2-variant, such as Mms2/Uev1A to specifically build ubiquitin chains. As described above, Mms2/Uev1A are the Lys63 linkage specificity determinant for Ubc13 catalytic activity (in the absence of a HECT E3). One study, however, suggested that Ubc13 could function with the RING E3 ligase RNF8, independent of the E2-variants [34]. This study used Mms2 deficient MEF cells, however the possibility of contributions from Uev1A were not ruled out. Additionally, the study performed siRNA knockdown of Mms2/Uev1A in HeLa cells where the western blot showed residual low expression of Mms2/Uev1A. Due to the possibility of residual Mms2 expression, our current more complete understanding of the mechanistic details of the RNF8/Ubc13/Mms2 complex, and the lack of other supporting studies, it is unlikely that Ubc13 functions without the E2-variants Mms2/Uev1A.

In general, one of many possible E3 ligases can bind the E2~Ub complex to recruit the charged E2 to the protein target. The E3 is not only a recruitment factor but plays an important role in stimulating the catalytic activity of the E2. The E3s TRAF6 [35, 36], Chfr [37], RNF8, RNF168 [33, 38], and the U-box E3 CHIP (carboxy terminus of Hsp70-interacting protein) [32, 39, 40] have all been shown to interact with Ubc13 and activate Ubc13 catalytic potential. The RING E3 stimulation ability is largely attributed to E2~Ub conformational selection from a more randomly distributed covalently linked ubiquitin [41], to a relatively confined, catalytically “active” position (Figure 1B, 1C) [39, 42–45]. Indeed, we recently demonstrated RING E3-mediated ubiquitin conformational selection through comparison of wild type and mutant RNF8 (Leu451Asp) in complex with Ubc13~Ub and Ubc13~Ub/Mms2 in solution using small-angle X-ray scattering (SAXS) [45]. The ubiquitin conformational selection is achieved through non-covalent interactions of the E2-linked ubiquitin molecule with E3 RING and E2 surfaces, which likely orients the E2-ubiquitin thioester linkage in the E2 active site to favor catalytic attack by the nucleophilic lysine residue. Additionally, we used the RNF8 Leu451Asp mutation to show that both the E2-stimulation and E2 recruitment of RING E3 ligases are critical for the cellular role of E3s.

## BLOCKING LYS63-LINKED UBIQUITIN CHAIN SYNTHESIS WITH OTUB1

**Figure 2:**
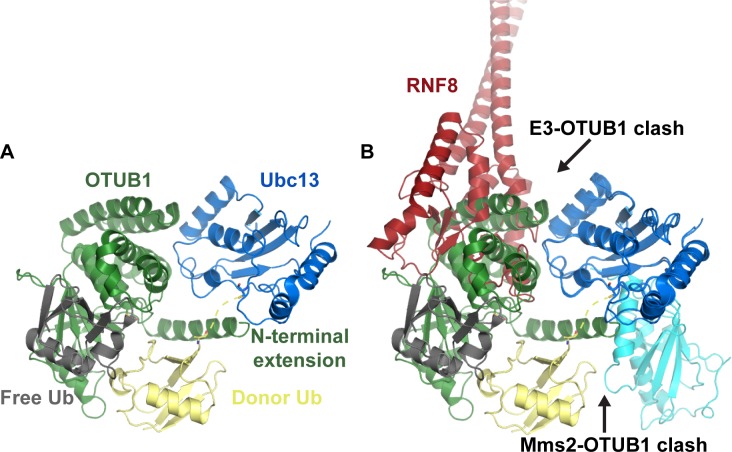
OTUB1 binds Ubc13~Ub to inhibit Lys63-linked ubiquitin chain formation **A.** Structure of OTUB1 bound to Ubc13~Ub with a free ubiquitin bound to OTUB1 (PDB: 4DHZ). **B.** OTUB1 Ubc13-binding overlaps with the RNF8 binding site and its N-terminal extension is predicted to interfere with Mms2 binding (PDB: 4ORH overlaid). OTUB1 is green, Ubc13 is blue, donor ubiquitin is yellow, free ubiquitin is gray.

OTUB1 is a deubiquitinase (DUB) isopeptidase that can cleave a ubiquitin-substrate isopeptide bond with specificity for Lys48-linked ubiquitin chains [46, 47]. It was found to negatively regulate chromatin ubiquitination at DNA double-strand break (DSB) sites by binding to Ubc13 and inhibiting its E2-conjugating activity [47]. OTUB1 knockdown caused persistence in both conjugated-ubiquitin (FK2) foci and 53BP1 foci [47]. The lowered OTUB1 levels also restored homologous recombination (HR)-mediated DSB repair in ATM-inhibited cells, monitored through a direct-repeat green fluorescent reporter (DR-GFP) assay that measures repair of an endonuclease site-specific DSB *via* HR [48, 49]. OTUB1 can also bind E2s of the UBE2E and UBE2D families [46, 47, 50]. Structures of OTUB1 with UbcH5b~Ub and Ubc13~Ub and free ubiquitin molecules and Ubc13/Mms2/OTUB1 have been determined [46, 51–53]. This work revealed that OTUB1 directly binds E2~Ub together with a second non-covalently bound ubiquitin and shed light on how OTUB1 inhibits a subset of E2s independent of its isopeptidase activity. Figure 2A shows the binding of a hybrid human (residues 1-45)/worm (OTU domain) OTUB1 to Ubc13~Ub. The hybrid was made because the important N-terminus of worm OTUB1 has poor conservation compared to human [52]. This N-terminal OTUB1 extension was shown to be necessary for E2 inhibition and interferes with the Mms2/Uev1A binding site on Ubc13 (Figure 2B) [52, 53].

The OTUB1 N-terminal extension also binds to the E2-linked donor ubiquitin in a similar manner to a UIM domain. The N-terminal extension shields the E2-ubiquitin linkage and prevents the donor ubiquitin interaction with the E2, which is important for its conjugation activity [46]. Interestingly, the free ubiquitin that binds to a distal site of OTUB1 in the structures was shown to greatly enhance OTUB1 binding affinity selectively towards conjugated Ubc13~Ub over free Ubc13 [46, 52]. The positions of the E2-linked donor and free ubiquitin in the OTUB1 structures resembles a Lys48-linked diubiquitin poised for isopeptidase deubiquitination where the hypothetical Lys48 linkage would be very close to the OTUB1 catalytic cysteine residue. Another obvious inhibitory feature of OTUB1 binding to Ubc13~Ub (or E2~Ub) is that it occludes/overlaps with the RING E3 binding site (Figure 2B).

## CATALYTIC AND STRUCTURAL CHARACTERISTICS OF UBC13

When considering the catalytic function of Ubc13, it is important to acknowledge that it requires interaction with either Mms2 in the nucleus, or Uev1A in the cytoplasm to form Lys63-linked ubiquitin chains. Ubc13 and Mms2 form a tight complex (K_D_ = 49 ± 7 nM [54]) and mutations that disrupt this complex have detrimental effects on Lys63-linked ubiquitin chain synthesis. In general, the catalytic rates of E2 enzymes are considered modest relative to other enzymes. To put Ubc13/Mms2 into perspective within the family of E2 enzymes a comparison of Ubc13/Mms2 to one of the fastest known E2s, the small ubiquitin-related modifier (SUMO) E2 enzyme Ubc9, showed that Ubc13/Mms2 has an approximate 14-fold slower *k_cat_* [30, 55].

**Figure 3:**
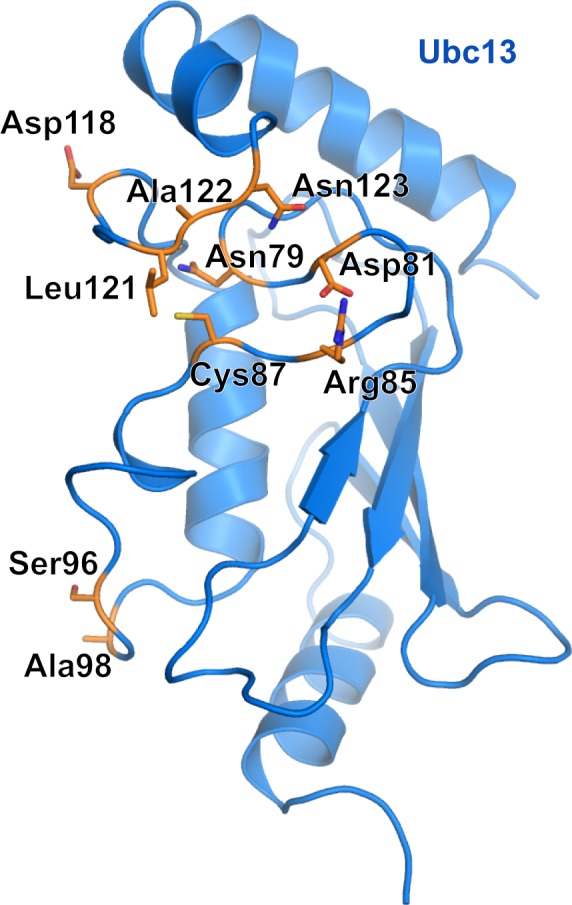
Important Ubc13 residues for catalytic activity Ubc13 residues that have been shown through mutation to affect Ubc13 ability to form ubiquitin chains. Important residues are highlighted orange and represented as sticks. Active site cysteine is also shown.

Several studies have examined residues important for the structural integrity and catalytic proficiency of Ubc13 (Figure 3). Berndsen et al. [56] made a series of mutations to Ubc13 Asn79, with varying effects on the catalytic efficiency of Ubc13. The mutations Asn79 to Ala or Asp decreased diubiquitin formation in the presence of Rad5 RING and caused a severe defect in diubiquitin formation in the absence of Rad5 RING (Figure 3). Three other Asn79 mutations, Asn79 to His, Ser, or Gln, decreased diubiquitin formation in the absence of Rad5 RING, but had normal diubiquitin formation in the presence of Rad5 RING. Collectively, the study by Berndsen et al. [56] demonstrated a structural role of Asn79 in Ubc13 catalytic function, in addition to its probable role in stabilization of the negative charge in the oxyanion thioester intermediate during nucleophilic attack by the incoming acceptor ubiquitin Lys63 [31]. We found that the Ubc13 mutations Ser96Asp and Ala98Asp of the conserved Ser-Pro-Ala motif resulted in loss of complex formation with the RNF8 RING dimer (Figure 3) [38]. In a separate study, we made a series of mutations to the Ubc13 active site loop to investigate the importance of dynamics to the catalytic function of the enzyme [57]. Ubc13 Asp118Gly or Ala122Gly caused different active site loop conformations than wild type, increased the loop flexibility on the pico- to nanosecond time scale, increased the rate of thioester hydrolysis, and impaired aminolysis. Ubc13 Leu121Gly had a similar active site loop conformation to wild type, a similar rate of thioester hydrolysis, and impaired aminolysis (Figure 3). Additionally, we mutated Ubc13 Leu121 to Ala, Val, or Ile, which resulted in an approximate linear increase in aminolysis rate with increasing availability of hydrophobic surface area that implicated Leu121 in desolvation of the active site [57]. In a study of Ubc13 inhibitors further discussed in a later section, we made a quadruple mutant of Ubc13 (Asp81Asn, Arg85Ser, Ala122Val, Asn123Pro), which flipped the Ubc13 active site loop to mimic the conformation present in UbcH5c, and this caused resistance to the small-molecule inhibitor NSC697923 (Figure 3) [27].

## PTMS THAT AFFECT UBC13 AND LYS63-LINKED UBIQUITIN CHAIN PRODUCTION

Cells often employ multiple layers of regulation on a given pathway frequently in the form of PTMs such as phosphorylation, ubiquitination, sumoylation, etc. Interestingly, a few PTMs have been discovered that exert their effect directly or indirectly on Ubc13. A recent study has examined Ser65-phosphorylated ubiquitin, which is produced by the protein kinase, PINK1 [58]. Ser65-phosphorylated ubiquitin activates the RBR E3 ligase Parkin and is involved in the onset of Parkinson's disease. Wauer et al. [58] tested a subset of E2 enzymes, *in vitro*, for ubiquitin charging (E2~Ub formation by E1) and polyubiquitin chain building. They found that all enzymes, including Ubc13, could be charged with ubiquitin, but that Ubc13/Uev1A-mediated Lys63-ubiquitin chain formation was inhibited by phospho-Ub. The authors hypothesize that the phosphate group on ubiquitin Ser65 would sterically preclude ubiquitin binding to Uev1A/Mms2. It remains to be determined whether phospho-Ub is used as an inhibitor of Ubc13 in a biological context, however the notion is intriguing and would add another layer of control to this critical, non-redundant enzyme.

Valimberti et al. [59] demonstrated that a conserved E2 Ser/Asp site exists in E2s that plays a role in correctly orienting the incoming substrate lysine toward the active site cysteine for catalysis. Some E2s are directly phosphorylated at the serine residue near the E2 active site loop, which increases the E2 catalytic activity [59]. Alternatively, other E2 enzymes conserve a negative charge at the same site near the active site loop and Ubc13 is one such E2 (Ubc13 residue Asp119).

Ubc13 can be targeted by another PTM termed ISGylation, where a ubiquitin-like protein, interferon-stimulated gene 15 kDa (ISG15) [60], is attached to a target lysine residue through its C-terminal glycine, much like ubiquitin [61, 62]. Cellular proteins are ISGylated upon interferon stimulation as part of an antiviral response [60]. The target site on Ubc13 is lysine 92, which is close to the active site cysteine. ISGylation of Lys92 was found to inhibit the ability of the E2 to be charged with ubiquitin by an E1 enzyme (Ubc13~Ub), but not the capacity to bind Mms2 [61, 62]. Minakawa et al. performed experiments where the components of the ISGylation system (UBE1L (E1), UbcH8 (E2) and ISG15), together with TRAF6 (to activate the NF-κB pathway) were transiently transfected into mammalian cell lines and NF-κB pathway activation was monitored using a luciferase reporter [60]. The NF-κB pathway was suppressed by the expression of the ISGylation system. In addition to the C-terminal glycine ISG15 modification, substrates can also be modified by the highly conserved and reactive ISG15 Cys78 residue, which is reducing agent-sensitive [63]. ISG15 Cys78 can form a disulfide bridge with the Ubc13 active site cysteine (Cys87), which would undoubtedly have an inhibitory effect on its catalytic function. Again, it is not yet known whether modification of Ubc13 Cys87 is physiologically relevant, nor whether the ISG15 disulfide modifications are relevant in general, so future studies must be done to interrogate these observations.

## CELLULAR SIGNALING PATHWAYS REGULATED BY UBC13 THAT MAINTAIN DNA INTEGRITY

### Role of Ubc13 in HR repair of DNA DSBs

**Figure 4:**
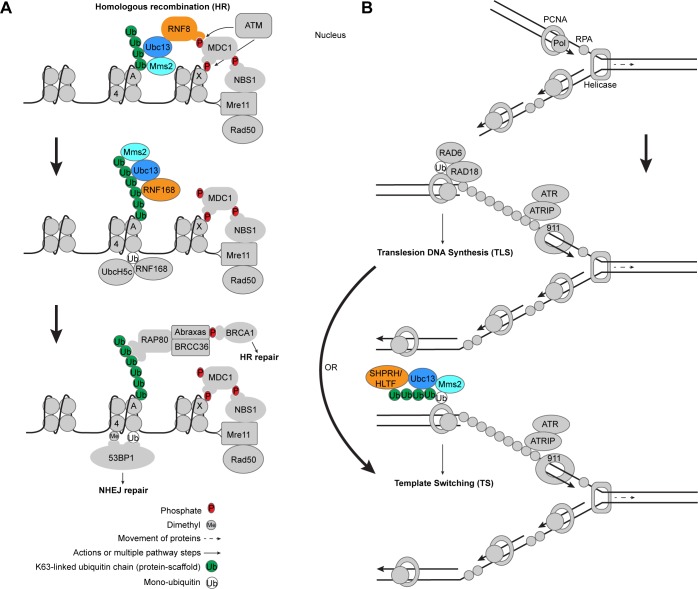
Ubc13 in DNA damage response and tolerance pathways **A.** Role of Ubc13 in DNA DSB signaling. DNA DSBs are initially recognized by the Mre11-Rad50-Nbs1 complex, leading to phosphorylation of chromatin associated proteins including H2AX (γH2AX), Nbs1 and MDC1. Ubc13/Mms2 participates with RNF8 to create Lys63-linked ubiquitin chains that recruit downstream repair factors for HR repair when the cell is in S/G2 phase. Histones H2AX, H2A, and H4 are labeled by letters X, A, and 4 in the nucleosomes, respectively. **B.** Role of Ubc13 in DNA replication stress. DNA template damage causes pausing of DNA polymerase, dissociation of the polymerase from the helicase, and accumulation of RPA on the resulting ssDNA (top and middle). The damage can be circumvented by the recruitment of a specialized polymerase that can read through the damaged template DNA (middle panel) or through a template switching mechanism that involves Ubc13 (bottom panel). Ubc13/Mms2 works with the E3 ligases SHPRH and HLTF to form Lys63-linked ubiquitin chains on previously monoubiquitinated PCNA to initiate TS repair at stalled DNA replication forks. The E3 ligases that participate with Ubc13 are colored orange.

Ubc13 functions in the response to DSBs in a cell cycle dependent manner [64]. During S or G2 phase the cell makes use of a newly replicated sister chromatid as an accurate template for repair of the damaged DNA. HR is initiated when the Mre11-Rad50-Nbs1 (MRN) complex binds the broken DNA ends to process them and recruits ataxia-telangiectasia mutated (ATM) kinase to phosphorylate the histone variant H2AX, termed ɣ-H2AX (Figure 4) [64–68]. ATM also phosphorylates a host of other DNA damage response (DDR) factors including CHK2 and p53, involved in cell cycle arrest, senescence, or apoptosis [69]. This provides a binding platform for the adaptor protein MDC1, which binds ɣH2AX through its BRCT domain [70–72]. MDC1 then undergoes two important phosphorylations [71]. The first is by casein kinase 2 (CK2), a kinase that constitutively phosphorylates Ser-Asp-Thr motifs on MDC1 that mediate binding interactions with Nbs1 (MRN nuclease complex component) [70]. The second is by the ATM kinase, which is bound by the FHA domain of the E3 ligase RNF8 (Figure 4) [70, 73, 74]. RNF8 binds the Ubc13/Mms2 E2 heterodimer with its RING domain to stimulate the formation of Lys63-linked ubiquitin chains [19, 38, 74–76]. Some studies suggest that a E3 ligase, HERC2, is sumoylated upon DSB induction and facilitates Ubc13 binding to RNF8 [77, 78]. It is likely that Ubc13/RNF8 then ubiquitinates previously monoubiquitinated H1-type linker histones, which recruits RNF168 through its motifs interacting with ubiquitin (MIUs) to amplify the Lys63-linked ubiquitin chains [79]. Once recruited, RNF168 can then function with the E2 UbcH5c to monoubiquitinate histones H2A/H2AX on Lys13-15 (Figure 4) [33, 67, 80]. The Lys15 ubiquitinated H2A (H2ALys15ub) then serves as a target for 53BP1 binding, which may actually promote NHEJ in opposition to BRCA1 [81]. The extended Lys63-linked ubiquitin chains generated from the cooperative activity of RNF8/RNF168 recruit RAP80 through its ubiquitin-interacting motifs (UIMs), which results in the binding of ABRA1 and BRCA1, to ultimately promote HR (Figure 4) [69, 82–85]. Multiple nucleases resect the broken DNA ends to form 3′ single-stranded overhangs, which are coated by the proteins RPA, RAD54, and RAD51 [86, 87]. With the help of RAD52, this nucleoprotein filament invades the homologous sister chromatid forming a D-loop, the 3′ overhang is extended by a polymerase, and the resulting Holliday junction is eventually resolved [88]. It should be noted that MDC1 was found to be Lys63-ubiquitinated in a Ubc13-dependent manner in the absence of DNA damage, and that this action facilitates RAP80 binding to MDC1 [89]. The possibility that these ubiquitination events play some role in the DNA damage response has not, however, been ruled out.

One understudied role of Ubc13 is in chromatin remodeling *via* acetylation and ubiquitination-dependent release of H2AX from IR-damaged DNA, which involves TIP60 and Ubc13 [90]. Additionally, a phosphoprotein nucleophosmin (NPM1) that moves between the cytoplasm and nucleus was reported to have a Ubc13-dependent late-stage role in HR repair, which also requires further investigation [91].

The nucleotide excision repair (NER) pathway deals with UV-induced DNA damage (for a review see [92]). One of the first major steps in global genome NER is the recognition of DNA-distorting UV lesions by a primary sensor, XPC, with the help of centrin 2 (CETN2) and UV excision repair protein RAD23 homologue B (RAD23B) [92]. In response to UV damage, XPC is poly-SUMO2/3 sumoylated, which provides a binding platform for the SUMO-interacting motifs (SIMs) of RING finger protein 111 (RNF111) [93]. Poulsen et al. show that RNF111 binds Ubc13/Mms2 and promotes Lys63 ubiquitination of sumoylated XPC, which regulates its accumulation on damaged DNA [93]. A further role for Ubc13 in UV-induced DNA repair comes from work which implicates Ubc13 and RNF8 in UV-induced ubiquitination [94]. Both proteins were found to localize to UV-induced DNA damage in HeLa cells, and Ubc13- and RNF8-knockdown resulted in increased sensitivity to UV, however not to the same extent as cells in which the NER endonucleases, XPG and XPF were knocked down. The siRNA-mediated knockdown of Ubc13 and RNF8 did not affect the DNA synthesis gap-filling stage of NER, suggesting that Ubc13/RNF8 plays a role peripheral to the predominant NER process [94]. 53BP1 is phosphorylated and accumulates at sites of DNA damage after UV irradiation [95], which was found to be partly dependent on RNF8 and Ubc13 through siRNA knockdown experiments [94, 95].

### DNA damage tolerance pathways and the role of Ubc13

When DNA replication machinery encounters DNA damage on the template strand, DNA damage tolerance (DDT) pathways are initiated to allow eukaryotic cells to continue replication past the damage [96, 97]. Two predominant DDT pathways are the error-prone translesion DNA synthesis (TLS) and error-free lesion bypass or template switching (TS) pathways (Figure 4) [96–98]. During DNA replication, proliferating cell nuclear antigen (PCNA) forms a ring around double-stranded DNA, which acts as a sliding clamp for DNA polymerases [97], while a helicase unwinds the DNA duplex. Upon DNA damage the E2 conjugating enzyme Rad6 and the E3 ligase Rad18 are recruited by replication protein A (RPA)-coated single strand DNA (ssDNA) [99, 100] to work together to monoubiquitinate PCNA on Lys164, which facilitates recruitment and interaction of PCNA with TLS polymerases (Figure 4) [101–103]. The ATR checkpoint is also engaged, which requires ATRIP and the 911 complex [100]. Elongation of monoubiquitinated PCNA with Lys63-linked ubiquitin chains by Ubc13/Mms2 with the E3 ligase Rad5 (yeast) transitions the DDT pathway to TS [104–107], using a newly replicated sister chromatid as an accurate template through DNA replication fork regression [96, 97]. The Ubc13-dependent Lys63-linked ubiquitin chains may be pre-formed and transferred as a unit to intermediate E2s and then to PCNA as opposed to extension of monoubiquitinated PCNA [108]. SHPRH and human helicase-like transcription factor (HLTF) are both human orthologs of yeast Rad5 with E3 ligase activities, which play a similar role to Rad5 in human cells (Figure 4) [109–112]. Alternatively, one study suggests that RNF8 may be able to act as the E3 ligase for Ubc13/Mms2 in Lys63-linked polyubiquitination of PCNA in the TS pathway [113]. TS may also involve a protein called TREX2, which appears to bind Ubc13 and to be important for PCNA ubiquitination [114]. Other studies have shown that yeast PCNA can be modified at Lys127/Lys164 with SUMO by Ubc9 [101], which may suppress undesirable HR [115–117], and may involve additional TLS and TS machinery [118].

### Role of Ubc13 in Fanconi anemia (FA) pathway

The FA pathway repairs DNA interstrand crosslinks (ICLs), which if left unrepaired, inhibit DNA transcription and replication, leading to stalled replication forks [119]. Upon DNA damage, two main protein complexes localize to the damaged DNA, which then signal downstream repair proteins. The first is a large complex of ~8 proteins called the FA core complex which monoubiquitinates the next complex in the pathway, the FANCI/FANCD2 complex. This complex further associates with FA protein complexes that include proteins such as BRCA2, BRIP1, and PALB2 [119]. The FA core protein complex FA-associated protein 20 kDa (FAAP20) was found to be necessary for normal DNA damage induced FANCI/FANCD2 monoubiquitination and FANCD2 recruitment to ICLs [120]. siRNA experiments demonstrated that recruitment of FAAP20 to DNA damage sites requires RNF8/Ubc13-dependent Lys63-linked polyubiquitination, and that FAAP20 can bind Lys63-ubiquitin chains *in vitro* with a ubiquitin binding domain (UBD) [120]. Further, mutation of the FAAP20 UBD abolished its accumulation at DNA damage induced ICLs.

### Ubc13 function at telomeres

Telomeres are the repetitive DNA sequences that together with the telomere-binding protein complex shelterin, protect the ends of chromosomes from premature shortening and inappropriate DDR protein recognition as DSBs [121]. The Tpp1 component of shelterin is required to bind and protect the DNA ends from the detrimental initiation of classical and alternative nonhomologous end-joining (NHEJ) repair pathways. Unscheduled NHEJ at telomeres can result in harmful chromosomal fusions, deletions, and translocations [121]. *In vitro* ubiquitination reactions demonstrated that the RNF8/Ubc13/Mms2 complex likely extends monoubiquitinated Tpp1 (Tpp1 was purified from 293T and E1 and RNF8 from Sf9 cells), with Lys63-linked ubiquitin chains [122]. In cells, RNF8 was shown to interact with Tpp1 through immunoprecipitation assays, suggesting that Ubc13 and RNF8 participate to Lys63-ubiquitinate pre-monoubiquitinated Tpp1. Additionally, siRNA knockdown of Ubc13 caused loss of Tpp1, likely through degradation [122]. Collectively, this study suggests that RNF8/Ubc13 Lys63-ubiquitinate and stabilize (pre-monoubiquitinated) Tpp1 to protect telomeres. As previously mentioned RNF8/Ubc13 participates in the recruitment of 53BP1 to sites of DNA DSBs, although this can be achieved in the absence of RNF8. 53BP1 represses DSB resection necessary for HR-repair at telomeres [121], so it is possible that RNF8/Ubc13 promotes 53BP1 recruitment to prevent telomere resection in addition to stabilizing Tpp1.

## ROLE OF UBC13 IN INFLAMMATION AND IMMUNE RESPONSE PATHWAYS

### NF-κB pathway

**Figure 5:**
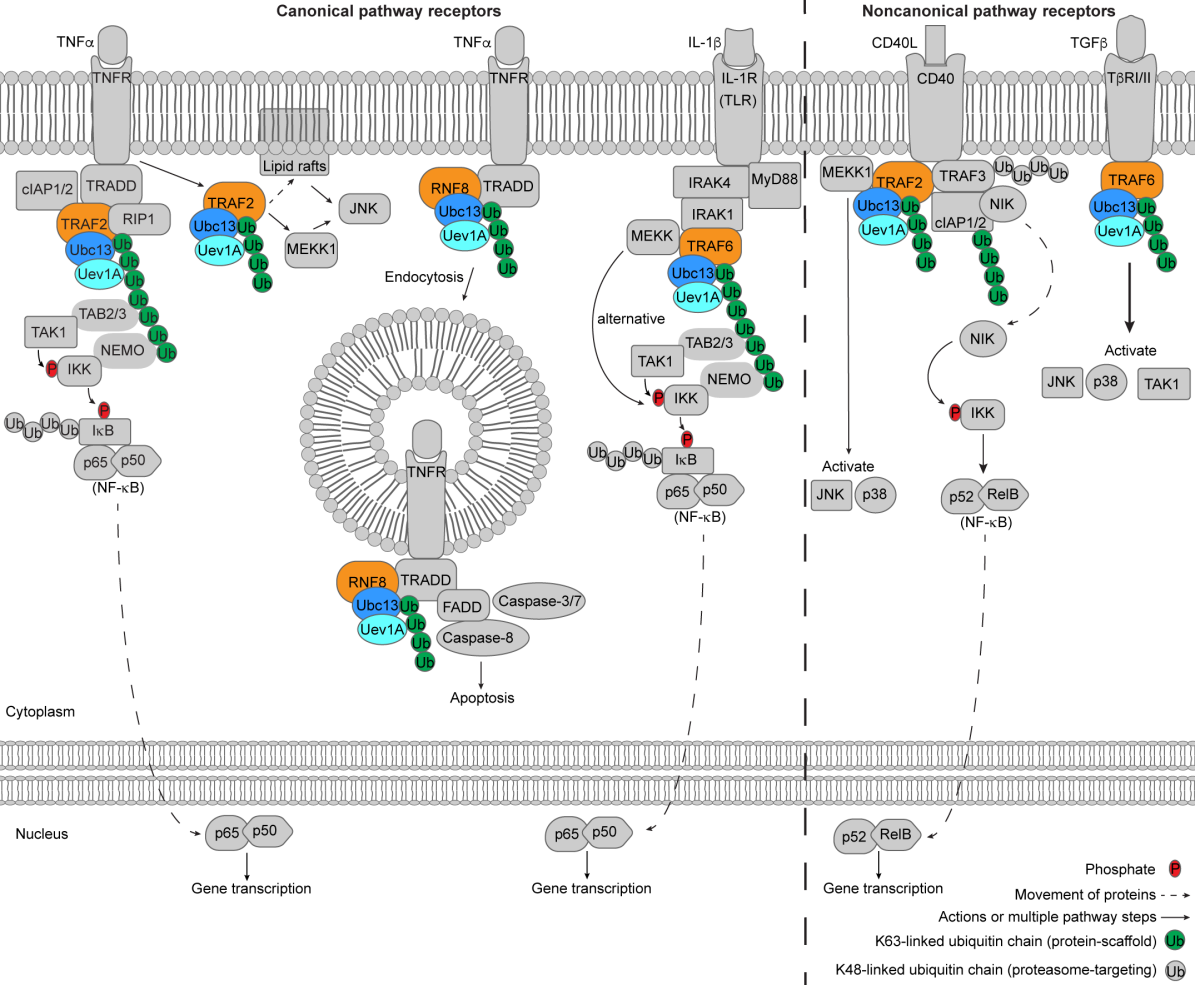
Ubc13 in immune and inflammation signaling In the canonical NF-κB pathway, receptor activation (left of dashed line), the TNFα stimulates TNFR in a Ubc13 dependent manner that either leads to NF-κB activation or apoptosis. IL-1β stimulation of IL-1R and stimulation of TLRs lead to NF-κB activation in a Ubc13-dependent way. In the noncanonical NF-κB pathway receptor activation (right of dashed line) CD40L stimulation of CD40 involves Ubc13-dependent Lys63-linked ubiquitin chains and leads to p52/RelB (NF-κB) activation. TGFβ stimulation of TβRI/II activates JNK, p38 and TAK1 through Ubc13-dependent activity. The E3 ligases that participate with Ubc13 are colored orange.

Ubc13 is heavily involved in inflammation and immune response pathways, largely through its role in NF-κB signaling (Figure 5). The NF-κB pathway [123–126] is a signal transduction network that is initiated by stimulation of a cell surface receptor and transduction of the signal through the cytoplasm, leading to activation and translocation of the NF-κB transcription factor into the nucleus [123, 127]. NF-κB signaling is grouped into canonical and non-canonical pathways, which all involve stimulation of a cell surface receptor by cytokines or pathogen-derived molecules. Bacterial lipopolysaccharide (LPS), tumor necrosis factor-α (TNFα), and interleukin-1β (IL-1β) stimulate the canonical NF-κB pathway, and BAFF, CD40L, and lymphotoxin-β heterotrimers (LTs) stimulate the non-canonical NF-κB pathway (Figure 5) [123, 126]. Cell surface receptor stimulation by TNFα and IL-1β in the canonical NF-κB pathway use different mechanisms to drive the same result: stimulation of phosphorylation of inhibitor of κB (IκB) by IκB kinase (IKK) and subsequent Lys48 ubiquitination-mediated degradation of IκB. The release of NF-κB from IκB allows NF-κB translocation to the nucleus [123, 126].

### Activation of canonical NF-κB pathway receptors

IL-1β stimulation of the canonical NF-κB pathway through binding to a Toll-like receptor (TLR) or IL-1 receptor (IL-1R) causes recruitment of the myeloid differentiation primary gene (MyD88) followed by IL-1β-associated kinases 1 (IRAK1) and 4 (IRAK4) to the intracellular receptor site (Figure 5) [126]. Pellino E3 ligases may participate with Ubc13 to polyubiquitinate (pre-monoubiquitinated) IRAK1 with Lys63-linked ubiquitin chains, to possibly contribute to IKK activation [128, 129]. TRAF6 is also recruited to the intracellular receptor and functions with Ubc13/Uev1A to form free Lys63-linked ubiquitin chains as well as TRAF6-conjugated chains [36, 124, 130–132]. TAK1 kinase is targeted to the Lys63-linked ubiquitin chains by TAB2/3, which allows TAK1 to phosphorylate and activate IKK (targeted to Lys63/Met1 hybrid chains by NEMO) (Figure 5) [124–126, 133, 134]. It should be noted that the type of ubiquitin chains (Lys63 [135–138] *versus* linear Met1 [139–143]) used to activate IKK was previously controversial, however the Emmerich et al. study [134] has provided clarity to the issue by demonstrating that the majority of linear Met1 ubiquitin chains are actually Lys63/Met1 hybrid chains, so it is likely that both chain types are necessary for proper IKK activation. A study by Yamazaki et al. [144] showed the existence of an alternative branched pathway of the IL-1β-induced NF-κB pathway, which diverges at the TRAF6 step. Mitogen-activated protein kinase (MAPK) kinase kinase (MEKK) binds to TRAF6 and may be ubiquitinated by TRAF6/Ubc13 although MEKK can be activated with a ligase-deficient RING mutant of TRAF6, which activates IKK [144]. It should be noted that the NF-κB pathway dependence on Ubc13 is cell type specific and a summary of Ubc13 knockout phenotypes can be found in the review by Wu and Karin [123].

In the TNFα-stimulated pathway, TNFα binding to a TNF receptor (TNFR), recruits a complex consisting of TNF receptor-associated protein with the death domain (TRADD) adaptor, receptor interacting protein-1 (RIP1), TNF receptor associated factor-2 (TRAF2), and cellular inhibitor of apoptosis proteins 1 and 2 (cIAP1/2) (Figure 5) [123, 125, 126]. RIP1 is Lys63-linked polyubiquitinated, possibly by Ubc13/Uev1A [145] and the E3 ligase TRAF2, but alternative types of polyubiquitin chains could play the same role in a Ubc13-independent manner [131]. NF-κB essential modulator (NEMO, also called IKKγ) and TAB2/3 bind the Lys63-linked ubiquitin chains to recruit the TAK1 and IKK kinases, leading to activation of IKK [125, 136, 137, 146]. Interestingly, an intracellular simulant of the NF-κB pathway called CC2D1A initiates the above mentioned Ubc13/TRAF2-dependent signaling similarly to TNFα receptor stimulation [147], however further studies are required to uncover possible upstream regulators of CC2D1A and provide a biological context for its function.

Receptor stimulation by TNFα may also initiate an IKK-independent signaling cascade, in which TRAF2 is ubiquitinated by Ubc13 (likely pre-monoubiquitinated), and translocated to insoluble cytoskeletal/membrane (lipid raft) areas, leading to activation of JNK but not NF-κB or p38 (Figure 5) [148]. Additionally, TNFR stimulation by TNFα has been reported to activate JNK (also known as SAPK), and germinal center kinase-related protein kinase (GCKR or MEKKK3) in a TRAF2/Ubc13-dependent manner, which may involve oligomerization and activation of MEKK1 [145].

TNFα can also bind the TNFR-1 receptor and signal an apoptotic response through Ubc13/RNF8-mediated Lys63-linked ubiquitination of TNFR-1, which causes internalization of the TNFR-1 receptor through endocytosis (Figure 5). Upon internalization of TNFR-1, the death-inducing signaling complex (DISC) is recruited, and caspase-3/7 and -8 are activated leading to apoptosis [149]. Results from UV stimulation of TNFR-1 support a regulatory role for Ubc13 in apoptotic signaling [150].

### Activation of noncanonical NF-κB pathway receptors

Ubc13 is also involved in activation of the MAPKs, JNK and p38. TNFR family receptor CD40 activation leads to formation of an intracellular complex that includes Ubc13, TRAF2, cIAP1/2, MEKK1, nuclear factor κβ-inducing kinase (NIK) and TRAF3 (Figure 5) [123, 151], which is stabilized by TRAF2/Ubc13-mediated Lys63-linked ubiquitination. TRAF3 is degraded *via* Lys48 ubiquitination-mediated proteasome targeting, which releases the complex in the cytoplasm that then activates JNK and p38 [151]. The transforming growth factor β (TGFβ) cytokine stimulates type I and II Ser/Thr kinase receptors (TβRI and TβRII) [152, 153], which initiates a Ubc13-dependent non-SMAD signaling pathway [123]. Similar to TNFR and IL-1R/TLR signaling, upon TGFβ receptor stimulation, Ubc13-dependent TRAF6 Lys63-linked auto-ubiquitination leads to TAK1 activation and the subsequent activation of MAP kinase kinases (MKK), which in turn activate p38 and JNK (Figure 5) [123, 154, 155].

### Role of Ubc13 in IL-17 receptor stimulation

A T helper 17 cell-produced proinflammatory cytokine, interleukin-17 (IL-17), is important for autoimmune diseases such as rheumatoid arthritis and multiple sclerosis and bacterial and fungal infections [156, 157]. IL-17 binds to the interleukin-17 receptor (IL-17R) A/C (IL-17RA/IL-17RC) complex to stimulate multiple pathways including the Act1-dependent pathway [156]. Act1 is a U-box E3 ligase shown to act with Ubc13/Uev1A to Lys63-linked ubiquitinate (likely pre-monoubiquitinated) TRAF6 upon IL-17R stimulation. The Act1-dependent Ubc13/Uev1A ubiquitination of TRAF6 leads to NF-κB activation. Liu et al. [157] showed that Act1-dependent Lys63-linked ubiquitination of TRAF6 is necessary for IL-17-mediated NF-κB activation, instead of TRAF6/Ubc13-mediated auto-ubiquitination.

### Involvement of Ubc13 in the negative regulation of immune signaling

Immune and pro-inflammatory pathways are finely tuned through the actions of immune repressors for appropriate innate immune responses. Signal transducer and activator of transcription 3 (STAT3) is one such repressor found to negatively regulate Ubc13 expression by the interleukin-6 (IL-6) cytokine, which is itself produced upon NF-κB-pathway induction as a negative feedback loop [158]. Receptor activator of nuclear factor κB (RANK) is a TNFR family member that is found on the cell surface of osteoclast progenitor cells, which relies on a Ubc13/TRAF6-dependent NF-κB response upon activation [159–161]. Stimulation of macrophages with IL-6 causes lowered Ubc13 mRNA and protein levels, and the repressive transcriptional relationship between STAT3 and Ubc13 was further demonstrated by chromatin immunoprecipitation, quantitative PCR, and mutational experiments [158]. In turn, antibody-mediated blocking of IL-6, with cell surface RANK stimulation *via* RANK ligand (RANKL) caused increased Ubc13 production in marrow-derived macrophages [158]. STAT3 signaling was shown to be further regulated by Ubc13-mediated Lys63-linked ubiquitination of an IKK subunit [162].

Myc-interacting zinc-finger protein 1 (Miz1) [163] and G protein pathway suppressor 2 (GPS2) [164] are proteins shown to play anti-inflammatory roles by antagonizing TNFα-stimulated Ubc13 association with a RING E3 ligase (TRAF2/6) to create Lys63-linked ubiquitin chains necessary for downstream JNK activation. Upon TNFα receptor stimulation, Miz1 is targeted for proteasomal degradation *via* Lys48 ubiquitination, but a Miz1 mutant unable to be tagged with Lys48 ubiquitin chains directly binds the TRAF2 RING domain and prevents the formation of a TRAF2/Ubc13 complex [163]. GPS2 is known as a transcriptional regulator, but also functions in the cytoplasm in a non-transcriptional capacity [164]. Yeast two-hybrid assays, GST pull-downs, and co-immunoprecipitation experiments demonstrated a direct interaction of GPS2 with TRAF2 and Ubc13, with a preference for ubiquitin conjugated Ubc13 (Ubc13~Ub). GPS2-Ubc13 interactions did not disrupt Ubc13 binding to Uev1A, but prevented *in vitro* TRAF2-dependent polyubiquitin chain formation [164]. Intriguingly, GPS2 could bind Ubc13~Ub in such a way as to prevent the previously mentioned conformational selection of the donor ubiquitin from occupying the activated position.

Another protein recently identified to be a negative regulator of the NF-κB pathway is A20 (TNFAIP3), which has both deubiquitinase (cleaves Lys63-linked ubiquitin chains [165]) and ubiquitination activities [166, 167]. As previously described, an important signaling step upon TLR or IL-1R stimulation *via* LPS or IL-1β is the association of Ubc13 with TRAF6 to make Lys63-linked polyubiquitin chains on TRAF6, which leads to the eventual activation of IKK and the translocation/activation of the NF-κB transcription factor. A20 works with an adaptor protein, Tax1 binding protein 1 (TAX1BP1), to bind TRAF6 and prevent its interaction with Ubc13 [166]. In addition to blocking Ubc13/TRAF6 binding, A20 facilitated Lys48-ubiquitination and degradation of Ubc13 in primary bone marrow-derived dendritic cells (BMDCs) and macrophages (BMDMs) after ~4-6 hours of IL-1 treatment [166].

### Role of Ubc13 in T cells

The adaptive immune response encompasses antibody producing B cells and a variety of T cell types [168]. T cell antigen receptors (TCRs) recognize major histocompatibility complex (MHC)-presented antigenic peptides on antigen presenting cells (APCs), which activate TCR signaling pathways that can include NF-κB activation and Ubc13 [168, 169]. Zhao et al. [170] characterized a RING E3 ligase called T cell RING protein identified in activation screen (TRAC-1), which has high expression in lymphoid tissues, appears to positively regulate T cell activation and can interact with Ubc13 to create Lys63-linked ubiquitin chains *in vitro*. T cells from mice with a mutated Ubc13 gene demonstrated deficient NF-κB and MAP kinase activation [171]. A type of T cell called regulatory T cell (T_reg_), suppresses the immune system to prevent unchecked immune function and inflammation through cytokine secretion and cell-cell contact [172]. Ubc13 conditional knockdown in T_reg_ cells caused the cells to acquire an effector phenotype that produced pro-inflammatory cytokines, which was shown to involve Ubc13-IKK signaling [173].

### Ubc13 role in IFN-γ production in natural killer (NK) cells

Another component of the innate immune system are natural killer (NK) cells, which can kill infected or transformed cells directly and can also secrete cytokines such as IFN-γ. Chen et al. identified an endoplasmic reticulum (ER) membrane protein called ER adaptor protein (ERAdP) that is constitutively expressed in NK cells and can activate NK cells likely through Ubc13-dependent NF-κB pathway activation. ERAdP directly interacts with Ubc13, as demonstrated through recombinant and cellular co-immunoprecipitations [174]. Ubc13 conjugation to ubiquitin (Ubc13~Ub) was enhanced in the presence of ERAdP, and Ubc13 was shown to be required for IFN-γ production. Together, these findings support a model in which ERAdP works through the Ubc13-NF-κB system to drive IFN-γ production in NK cells.

### Ubc13 in cytoplasmic bacterial infection sensing by NOD2

Nucleotide-binding oligomerization domain-containing protein 2 (NOD2) is an intracellular, cytoplasmic sensor of bacterial infection, specifically binding muramyl dipeptide (MDP) on bacterial peptidoglycans [175, 176]. NOD2 single nucleotide polymorphisms (SNPs) are associated with Blau syndrome and Crohn's disease, which are inflammatory disorders [176, 177]. From an auto-inhibited state, NOD2 is activated by binding to MDP, which then binds RIP2, effectively disrupting a RIP2/MEKK4 complex [176]. RIP2 is polyubiquitinated in a Ubc13/TRAF6 dependent manner [178], and the subsequent recruitment of TAK1 leads to NF-κB activation [176]. Additionally, other E3 ligases may be able to functionally substitute for TRAF6 [177].

### Ubc13 involvement in implant inflammation

An unfortunate stimulant of inflammatory pathways such as the NF-κB pathway are polymethylmethacrylate (PMMA) particles, which are shed from implants and cause inflammatory osteolysis [179]. PMMA particles increase inflammation by TAK1 activation, with the induction of Ubc13 and TAK1 binding to NEMO, along with TRAF6 binding to NEMO, suggests a route for NF-κB pathway activation.

### Ubc13 function in response to hypoxia

Hypoxia induces NF-κB activation through a signaling cascade that is Ubc13-dependent [180], but is initiated differently than those described earlier for the NF-κB pathway. Culver et al. [180] demonstrated that NF-κB activation *via* hypoxia requires calcium/calmodulin-dependent kinase 2 (CaMK2) and the presence of calcium (Ca^2+^), which leads to TAK1/IKK/NF-κB activation. Interestingly, hypoxia-induced NF-κB activation required Ubc13, but not TRAF2/6, however XIAP (X-linked inhibitor of apoptosis protein) [181] is a candidate E3 ligase that could possibly function with Ubc13 in this pathway [182]. Further, hypoxia did not lead to canonical NF-κB pathway IκB degradation, but instead resulted in increased sumoylation of IκB Lys21, which prevented its degradation [180]. Interestingly, the sumoylation of IκB seems to increase release of RelA (an NF-κB subunit) suggesting an alternative mode of NF-κB activation, which was demonstrated through desumoylation enzyme depletion experiments. One percent oxygen was used as a hypoxic condition in the Culver et al. experiments. It should be noted that many cell culture experiments are performed at atmospheric oxygen levels of ~21%, however cellular oxygen levels within a mammal are estimated to be closer to 5% (range of ~1-11%). This difference may have important consequences for oxidative metabolism and experimental results in at least lymphocytes and neurons [183–185]. Hydroxylation of Ubc13 may also play a role in hypoxic IL-1β-induced signaling, but further direct evidence of regulatory importance of this modification is required [186].

## CROSSTALK BETWEEN THE DDR AND NF-κB PATHWAYS INVOLVES UBC13

Mechanistic insight into how DNA damage activates the NF-κB transcription factor has been uncovered [187, 188]. In 2010, two simultaneously published studies showed major roles of the DDR regulatory kinase ATM and Ubc13 in DDR-dependent activation of NF-κB, although the details of signaling differs between the two studies. Hinz et al. showed that DNA damage induces two parallel signaling streams that converge into one in the cytoplasm to activate NF-κB [187]. Upon DNA damage ATM [189–191] and poly(ADP-ribose)-polymerase-1 (PARP-1) [192–195] are recruited to DNA DSBs. In one DDR-dependent NF-κB activation stream DNA damage-activated ATM translocates from the nucleus to the cytoplasm, which was abolished in the presence of a calcium chelator, indicating calcium dependence [187]. It was demonstrated through pull-down experiments in cell extracts that ATM contains a conserved motif that directly binds endogenous TRAF6 and promotes its polyubiquitination *via* Ubc13. Polyubiquitinated TRAF6 subsequently recruits cIAP1, which causes NEMO monoubiquitination, and TAB2-dependent phosphorylation of TAK1 leading to downstream IKK-NF-κB activation. In the other stream of DDR-dependent NF-κB activation, nuclear PARP-1 synthesis of poly(ADP-ribose) (PAR) chains induces complex formation of ATM, NEMO, and the SUMO1 E3 ligase PIAS4 (or PIASy) [196], which results in sumoylation and possibly phosphorylation of NEMO [187]. Interestingly, the sumoylated NEMO translocates to the cytoplasm and acts as the substrate for the previously mentioned NEMO monoubiquitination, which eventually leads to downstream NF-κB activation.

The second study that showed a major role of the DDR regulatory kinase ATM and Ubc13 in DDR-dependent activation of NF-κB also involves NEMO, TAK1, TAB2/3, IKK, XIAP, and a protein rich in glutamate, leucine, lysine, and serine (ELKS) [188]. Wu et al. demonstrated that upon DNA damage, ATM phosphorylates NEMO Ser85, which results in a Ubc13, Lys63-linked ubiquitin chain, TAB2/3-dependent ELKS/TAK1 complex that promotes IKK activation. NEMO is required for TAK1 activation and its polyubiquitin binding is required for activation of IKK, but not of TAK1. ELKS is also Lys63-linked ubiquitinated, which is facilitated by the E3 ligase XIAP, and likely Ubc13. Collectively, the Wu et al. results describe components of an ATM- and Ubc13-dependent signaling cascade that activates the NF-κB transcription factor in response to DNA damage [188].

In one instance of DDR-NF-κB pathway crosstalk, a factor involved in the NF-κB pathway was shown to be involved in the DDR. A protein called B-cell lymphoma/leukemia 10 (BCL10) participates in the activation of the NF-κB pathway in B and T cells, which involves TRAF6/Ubc13/Uev1A-mediated Lys63-linked ubiquitination of NEMO and subsequent activation of TAK1-IKK and NF-κB [197–199]. BCL10 was recently shown to be phosphorylated by ATM in response to DNA damage, to colocalize with DNA damage dependent ɣH2AX foci and to participate in HR repair factor recruitment to sites of damage [197, 200]. BCL10 also associates with RNF8, and this seems to be partially dependent on the RNF8 FHA domain. The authors offer the suggestion that RNF8 FHA may bind to BCL10 (Thr91) TQXF motif (known to be targets for phosphorylation) [197]. RNF8 ubiquitinates BCL10 predominantly with Lys63-linked ubiquitin chains, which requires the previously mentioned BCL10 phosphorylation and likely pre-monoubiquitination. Zhao et al. found that upon siRNA-mediated knockdown of BCL10 expression, the binding of RNF8 and Ubc13 was diminished. They conclude that “BCL10 presents Ubc13 to RNF8,” however the fact that Ubc13 and RNF8 efficiently bind *in vitro*, suggests that perhaps BCL10 disrupts association of either Ubc13 or RNF8 with another factor, which allows their subsequent binding.

## ROLE OF UBC13 IN DIVERSE CELLULAR SIGNALING

### Ubc13 role in the regulation of p53

A few studies have reported an interesting protein-protein interaction between Ubc13 and p53 involved in the DDR and transcription/translation regulation. The distribution of p53 in the cell depends on the level of differentiation, where p53 is largely cytoplasmic in undifferentiated cells and many cancer cell lines, while many differentiated cell lines exhibit predominantly nuclear/perinuclear p53 [201–207]. Laine et al. showed that Ubc13 regulates the subcellular distribution of p53 through its ubiquitination activity [208]. They uncovered a direct interaction between the C-terminus of p53 and Ubc13 that requires either Mms2 or Uev1A (i.e. the functional E2 heterodimer) and this interaction has also been observed in a zebrafish system [209]. The Mdm2-mediated polyubiquitination and subsequent proteasomal degradation of p53 is suppressed by Lys63-linked ubiquitination *via* Ubc13, although mono-/di-ubiquitination of p53 is unaffected by Ubc13. This Ubc13-dependent ubiquitination also prevents p53 tetramerization, attenuates its transcriptional activity, and localizes it to the cytoplasm [208]. Interestingly, these Ubc13-dependent changes to the state of p53 are diminished upon IR, which may reflect the need for their individual participation in the DDR. This is also likely due, in part, to p53-mediated downregulation of Ubc13 expression upon DNA damage [208]. The same group also found a p53/Ubc13 association on actively translating polysomes [210].

Interestingly, Solozobova et al. found that embryonic stem cell (ES) lines have high p53 expression compared to a differentiated cell line, mouse embryonic fibroblasts (MEFs) [206], but with a lower half-life, indicating decreased protein stability. siRNA knockdown of Ubc13 caused a reduction in Mdm2 and p53 amounts in ES cells, establishing a functional relationship between Ubc13 and p53 in ES cells.

### Hematopoiesis involves Ubc13 activity

Ubc13 has been shown to play a role in hematopoiesis [211, 212]. Wu et al. [211] generated Ubc13 conditional knockout mice, as Ubc13 knockout was embryonic lethal [213]. Ubc13 deficiency had a serious effect on the bone marrow, mesenteric lymph nodes, thymus and spleen which affected the production of platelets, and white (WBC) and red blood cells (RBC). Due to an established role of Wnt signaling in hematopoiesis [214, 215], Wu et al. investigated important steps in Wnt signaling such as the stabilization of β-catenin and subsequent transcription of the Wnt target genes, Lef1, Ccnd1, Tcf1, and Axin2 [211]. Elevated expression of the Wnt target genes and stabilized β-catenin suggested a negative regulatory role for Ubc13 in Wnt signaling that has important implications for hematopoiesis.

Triad1 (2 RING fingers and DRIL (double RING finger linked) 1) is a primarily nuclear RING-between-RING (RBR) E3 ligase that uses an active site cysteine and RING domains to transfer ubiquitin to a substrate [7]. Triad1 functions in the inhibition of clonogenic growth and subsequent maturation of immature blood cells into monocytes and granulocytes [212, 216]. Co-immunoprecipitations in human cells and *in vitro* surface plasmon resonance (SPR) experiments demonstrated Triad1 binding to Ubc13 and Ubc13/Mms2, respectively. Interestingly, the RING1 domain of Triad1 is necessary for interaction with UbcH7 to form Lys48-linked ubiquitin chains, whereas RING2 is needed for interaction with Ubc13 to form Lys63-linked ubiquitin chains [212]. Both RING1 and RING2 are required for Triad1 function in blood cell differentiation, and both Lys48- and Lys63-linked polyubiquitin are found in these cells dependent on Triad1. Taken together, this study suggests that the ability of the Triad1 E3 to drive formation of both kinds of polyubiquitin is critical for its role in the control of myeloid proliferation.

### Role of Ubc13 in ER-associated degradation (ERAD)

The ERAD system is part of an important cellular process that employs ubiquitination-dependent proteolysis to deal with unfolded/misfolded proteins. RING finger protein 5 (RNF5) is an ER-anchored E3 ligase that regulates misfolded protein degradation. JNK-associated membrane protein (JAMP) facilitates and increases ERAD through association with proteasome components such as gp78 and p97 [217]. RNF5 regulates the response to misfolded/unfolded proteins by functioning with Ubc13 to form Lys63-linked ubiquitin chains on JAMP, which prevents JAMP association with the proteasomal and ERAD components, Rpt4/5/6 and p97, respectively; this in turn diminishes the ability of JAMP to act as a proteasomal scaffold [217].

### Ubc13 in neural development and neurodegenerative diseases

Early studies demonstrated a role for the *Drosophila* ortholog of Ubc13, Bendless (Ben), in neural development [218–223]. Ben is necessary for synaptic transmission between the giant fiber neuron and both the tergotrochanteral muscle (TTM) [222] and tibial levator muscle (TLM) motor neurons [223]. Further, flies lacking Ben showed abnormal thoracic muscle organization, decreased mobility of newly hatched flies, and increased mortality in late pupal stages [221]. A study showing Ben mutant defects in the visual system and increased Ben RNA transcript expression in the *Drosophila* nervous system during embryo development, suggested a prominent role for Ben in early neural development [218]. Ben was demonstrated to be essential for the pre-synaptic initiation of synapse formation for subsequent synaptic growth and development [224]. Ben was shown to be important for long-term memory, which suggested a post-developmental role of Ben in the nervous system [225].

Ubc13 is involved in two neurodegenerative diseases characterized by protein misfolding, Huntington's disease (HD) [226] and Parkinson's disease (PD) [227–230]. The primary feature of HD is the variable DNA repeat expansion of CAG in the huntingtin (HTT) gene [231, 232], which translates into a huntingtin protein (or fragment) with a lengthened N-terminal polyglutamine region prone to aggregation in neurons and accumulation in synaptic regions [233]. In their study, Yin et al. examined the brain tissues of rhesus monkeys using a cellular fractionation technique that isolates synaptosomes [226], which are artificial vesicles formed through centrifugal-induced severing of the axon terminals from neurons that comprise the synaptic components [234]. Ubiquitin-dependent proteasomal activity in brain tissues, but not the muscle or liver, decreased with the age of monkey, while there was an increase in ubiquitin-conjugates [226]. There was also an age-dependent increase in Ubc13 expression in monkey brain tissues, but not muscle or liver tissues. Interestingly, experiments in which Ubc13 siRNA and huntingtin mutant constructs were co-transfected into cultured cells demonstrated that inhibition of Ubc13 expression caused a decrease in mutant huntingtin aggregates in human HEK293 and rat PC12 cells. Immunoprecipitations in transfected cells showed that one of the mutant huntingtin constructs (exon 1-97Q) was more heavily ubiquitinated with Lys63-linked chains than Lys48. The authors also found that shRNA silencing of Ubc13 in HD model mice (brains) significantly decreased aggregation of full-length mutant huntingtin [226]. Collectively, this data suggests that Ubc13 may play an important role in huntingtin accumulation in neurons and neurotoxicity in HD.

Predominant features of PD include the presence of protein-rich inclusions in neurons called Lewy bodies, the decline in dopaminergic neurons in the brain [235], and dysfunctional mitochondria [227]. Parkin is a RBR E3 ligase involved in the pathogenesis of PD [236], which is thought to be related to the lysosome-autophagy pathway [235]. Autophagy is a process that isolates a section of the cytoplasm in a double membrane vesicle called the autophagosome, that then fuses with the lysosome, unloading the enveloped contents into the acidic lysosome environment for degradation [237]. Tan et al. demonstrated that Lys63-linked ubiquitination promotes the formation of protein aggregates/inclusions in a human cell line and that Lys63-linked ubiquitin aggregates are preferentially targeted for autophagy [238]. Further, Lim et al. has shown that Ubc13 is recruited to function with Parkin and the degree of Lys63-linked polyubiquitination is increased when the proteasome is inhibited under conditions of proteolytic stress [230]. Additionally, proteasome inhibition of Ubc13 knockout MEFs compared to wild type suggests a Ubc13-mediated protective function for cells during proteolytic stress. A likely target of Ubc13/Parkin-mediated lysosome-autophagy is the DUB ubiquitin C-terminal hydrolase L1 (UCH-L1), which is itself important for neurological function, as mutations in UCH-L1 cause neurodegenerative disorders [228].

Parkin is involved in mitophagy, a process in which mitochondria that have been damaged are selected for removal [239]. Previous hypotheses that Ubc13/Parkin-mediated Lys63-linked ubiquitin chains on damaged/stressed mitochondria promoted mitophagy have been shown to be untrue [229], and instead these chains appear to promote mitochondrial fusions (mitofusions) [227].

### MicroRNA regulation of Ubc13

Small non-coding RNAs called micro-RNAs (miRNAs) have previously been shown to regulate gene expression and to be involved in radio- and drug-resistance of cancer cells [240, 241]. Zhang et al. showed that a miRNA, miR-205, represses the expression of Ubc13 and the transcription factor zinc finger E-box binding homeobox1 (ZEB1), which effectively impedes the DDR [241]. In response to ionizing radiation (IR), tumor cells downregulate miR-205 expression [242]. Downregulation of miR-205 in response to IR would increase tumor radioresistance due to enhanced DNA repair. Zhang et al. demonstrated the therapeutic potential of miR-205, through nanoliposomal delivery of miR-205 to radioresistant tumor cells and xenograft tumors, which had a significant radiosensitizing effect [241].

### Role of Ubc13 in thymidine synthesis

The folate-dependent biosynthesis of thymidine nucleotide is important for cell growth and replication, and depletion of thymidine triphosphate (TTP) pools results in genome instability [243, 244]. The input material for synthesis of thymidine monophosphate (TMP) is methylenetetrahydrofolate (methyleneTHF), which is used by the enzyme thymidylate synthase to convert deoxyuridine monophosphate (dUMP) into deoxythymidine monophosphate (dTMP) [243]. Serine hydroxymethyltransferase (SHMT) is the enzyme that generates methyleneTHF from THF and serine. The regeneration of THF uses NADPH and dihydrofolate reductase (DHFR). Ubc13 is involved in the regulation of SHMT1 through Lys63-linked ubiquitination and subsequent nuclear export of SHMT1, which leads to degradation of SHMT1 in the cytoplasm [243, 245]. The Ubc13-dependent Lys63-linked ubiquitin modification of SHMT1 may also compete with SUMO-dependent degradation of SHMT1 in the nucleus [243].

### Ubc13 involvement in mitotic checkpoints

Ubc13 functions with the RING E3 ligase, checkpoint with FHA and RING domains (Chfr), in an early mitosis checkpoint pathway [37, 246, 247]. In 2000, Scolnick and Halazonetis uncovered a Chfr-dependent pathway in human cell lines that delays mitotic transition from prophase to metaphase under mitotic stress and found that Chfr is mutated or not expressed in four cancer cell lines [246]. The same research group later found that Chfr functions with Ubc13/Mms2 to make Lys63-linked ubiquitin chains *in vitro* [37]. More recently, genetic evidence has suggested that in yeast Ubc13 may function with Chfr homologs to delay the cell cycle, suggesting conservation of this pathway [247].

### Ubc13 participation in growth hormone signaling

Growth hormone (GH) signaling is critical to adolescent longitudinal bone growth, bone mass, maturation of the skeletal system, and maintenance of bone mass in adults [248]. The 191 amino acid GH peptide binds to a class I cytokine receptor called growth hormone receptor (GHR) [248, 249]. Cells can regulate their sensitivity to GH by controlling the amount of cell surface GHRs through endocytosis and subsequent lysosomal degradation of GHRs [250]. Slotman et al. demonstrated that U-box E3 CHIP binds GHR and likely works with Ubc13 to form Lys63-linked ubiquitin chains, which play a role in proper signaling for GHR endocytosis. Both Ubc13 and CHIP were shown to be necessary for GHR endocytosis [250].

### Involvement of arabidopsis Ubc13 in auxin signaling

Ubc13/Uev1A is conserved in plants with a role in DNA damage tolerance/repair [251, 252] and root development [253]. Auxin is a small molecule plant hormone that is important for root development and organization [254]. A double mutant of the Arabidopsis Ubc13 genes demonstrated root development defects including shorter primary roots, and reduced number of lateral roots, and root-hair density [253]. Wen et al. compared endogenous levels of auxin in wild type and Ubc13 null plants and found the mutant to contain about half the amount of auxin. Treatment of wild type and Ubc13 mutant plants with a synthetic auxin [255], α-naphthalene acetic acid (NAA), revealed the response to auxin and subsequent root development to be dependent on Ubc13 [253].

### Role of Ubc13 in sodium/potassium pump regulation

Ubc13 is involved in the regulation of the sodium/potassium ATPase (Na^+^/K^+^ ATPase), a plasma membrane-bound ATP-driven ion transporter that transports Na^+^ and K^+^ against their concentration gradients to form an electrochemical gradient across the plasma membrane [256]. The proper balance of sodium/potassium ions is critical to a plethora of fundamental cellular processes, such as electrical excitability, nutrient uptake, pH, and regulation of cell volume, among others. Protein-protein interactions between the Na^+^/K^+^ ATPase and multiple intracellular partners regulate diverse cellular signaling pathways (reviewed by Reinhard et al. [256]). Hoxhak et al. [257] found that Ubc13 interacts with two RING E3 ligases, ZNRF1 and ZNRF2 (ZNRF1/2) and that ZNRF1/2 stimulates Ubc13 Lys63-linked ubiquitination activity. Na^+^/K^+^ ATPase is composed of an α- and β-subunit, for which the α-subunit is known to mediate many protein-protein interactions [256]. ZNRF1/2 can interact with the Na^+^/K^+^ ATPase α-subunit through their ubiquitin-binding UBZ domains, however mutation of the UBZ zinc-binding cysteines to alanine did not abolish ZNRF1 interaction with Na^+^/K^+^ ATPase α-subunit, suggesting this interaction is not mediated by ubiquitin. The role of ZNRF1/2 is likely related to the signaling involved in the regulation of the levels of Na^+^/K^+^ ATPase in the plasma membrane, as siRNA-mediated knockdown of ZNRF2 prevented ouabain (a Na^+^/K^+^ ATPase inhibitor) from reducing Na^+^/K^+^ ATPase levels in the plasma membrane [257]. Collectively, the results suggest Ubc13 may play a role in the regulation of Na^+^/K^+^ ATPase through intracellular signaling activities, but direct evidence of Ubc13 involvement is still required [257].

### Possible involvement of Ubc13 in regulation of cell motility

Focal adhesions are major points of contact between cells and the external framework that stabilizes them, the extracellular matrix (ECM) [258, 259]. Paxillin is an adaptor that mediates crosstalk between signaling proteins and the cytoskeleton [258], and is involved in signaling at focal adhesions necessary for cell motility [259]. Didier et al. established the RING E3 RNF5 as a mediator of paxillin localization, demonstrating that RNF5 targets paxillin for ubiquitination, which decreases the presence of paxillin at focal adhesions and increases its cytoplasmic concentration without increasing paxillin degradation [259]. Due to a lack of RNF5-induced paxillin degradation, Ubc13 was hypothesized to work with RNF5 to create Lys63-linked ubiquitin chains, and this ability was confirmed *in vitro*. Expression of an inactive mutant of Ubc13 in human cells inhibited RNF5-mediated ubiquitination, which in combination with a lack of RNF5-mediated degradation led Didier et al. to suggest that Ubc13 likely works with RNF5 to target paxillin for ubiquitination [259]. Additionally, RNF5 was found to affect cell motility in paxillin-null cells, which suggested that RNF5 may be involved in targeting other cell motility-related proteins. Further studies are required to provide direct evidence for a role of Ubc13 in cell motility.

### Ubc13 in fertilization and spermatogenesis

Ubc13 plays a role in sexual reproduction, specifically spermatogenesis and fertilization. During meiosis there is exchange of genetic information between homologous chromosomes through meiotic recombination, which requires the generation of numerous DSBs that are repaired after recombination [260]. Due to little homology between the X and Y sex chromosomes, there is an extended DNA repair period with prolonged presence of DNA damage repair proteins in the largely unsynapsed XY body structure [260]. There was little to no detection of Ubc13 at XY body DNA structures in male mice during spermatogenesis, suggesting Ubc13 may not be involved in DNA repair of the induced DSBs [261]. However in a separate study Ubc13 mRNA transcripts were shown to be highly expressed in mouse testes [262]. Androgen suppression caused DNA damage through oxidative stress and proteomics studies identified Ubc13 as a gene among those upregulated in male rat meiotic cells in response to androgen suppression. Increased Ubc13 in oxidative stress conditions in the testes likely reflects the role of Ubc13 in DNA repair pathways such as template switching (Figure 4) [263]. Endocytosis of maternal membrane proteins upon egg fertilization and subsequent late endosome sorting may involve Ubc13-mediated Lys63-linked ubiquitination in humans, as shown in *Caenorhabditis elegans* [264].

Several consecutive miscarriages prior to twenty weeks of gestation warrants a recurrent miscarriage (RM) diagnosis [265]. As previously discussed Ubc13 is important for T_reg_ cell-mediated suppression of inappropriate immune responses. Upon fertilization, the zygote contains both maternal and paternal DNA, but is hosted in the mother's body, whose immune system therefore must quickly build a tolerance for the male-derived antigens of the zygote [265]. Liu et al. examined the frequency of T_reg_ cells with sperm antigen specificity (^SAS^T_reg_) for their husbands' sperm in women with RM, which would be indicative of the female immune tolerance to the male's antigens [265]. RM women expressed less Ubc13 mRNA and protein, and had an overall lower frequency of ^SAS^T_reg_ cells than the control group [265]. Consistent with previous results [173], Liu et al. found that Ubc13 knockdown induced T_reg_ cells to acquire effector T cell functions. The lowered Ubc13 expression in RM women would therefore be expected to have a negative effect on maternal immune tolerance for the paternal sperm.

## ROLES OF UBC13 IN DIVERSE CELLULAR INFECTIONS

### Involvement of Ubc13 in the response to viral infection

TRIM5 is a RING E3 ubiquitin ligase host cell protein that is known to play a role in restricting infection by retroviruses after they have entered the cell cytoplasm, which includes the human immunodeficiency virus (HIV)-1 [266–269]. TRIM5 promotes innate immune signaling pathways, including the previously discussed NF-κB pathway [267]. TRIM5 oligomerizes and recognizes retroviral capsids, and participates with Ubc13/Uev1A to form free Lys63-linked ubiquitin chains in the cytoplasm, which activate TAK1, resulting in the subsequent activation of the NF-κB and AP-1 transcription factors [267]. TRIM5 likely uses the previously discussed E3-dependent conformational selection to stimulate Ubc13~Ub/Uev1A E2 activity, and this is dependent on TRIM5 dimerization, which is facilitated by retroviral capsid binding [269].

### Viral proteins hijack Ubc13 upon infection

Adult T-cell leukemia/lymphoma (ATLL) is a disease that is caused by human T-cell leukemia virus type 1 (HTLV-1) that develops after a long 40-60 year latent period in three to five percent of people infected [270]. HTLV-1 targets T lymphocytes (CD4^+^), but can also infect myeloid and dendritic cells leading to HTLV-1 associated myelopathy/tropical spastic paraparesis. The major HTLV oncoprotein, Tax, hijacks host cell signaling pathways, including the NF-κB pathway [270]. Shembade et al. determined that Tax is primarily Lys63-linked ubiquitinated and also performed immunoprecipitation assays in cells expressing Tax to demonstrate that Tax binds Ubc13, an interaction necessary to facilitate Tax binding to NEMO [271]. Ultimately, the Tax-dependent Lys63-linked ubiquitin chains formed by Ubc13 are used to aggregate signaling proteins and activate TAK1 and the IKK complex [272, 273]. Additionally, Tax-mediated Ubc13 activation may involve the RING E3 ligase, RNF8 [273].

Many viruses use a strategy to evade the immune systems of host organisms whereby viral signaling proteins stop the host cell from presenting viral components on the host cell surface through the major histocompatibility complex class I (MHC I) molecules. The MHC I-presented viral components are recognized by the host cytotoxic T (T_c_) cells, which targets the infected cells for extermination [274, 275]. Kaposi's sarcoma herpes virus (KSHV) causes Kaposi's sarcoma cancer and employs a ubiquitin E3 ligase K3, which contains a RING-CH domain similar to classical RING domains, to target MHC I molecules on the host cell plasma membrane for endocytosis and lysosomal degradation [275, 276]. Duncan et al. demonstrated that K3 binds UbcH5b/c to first monoubiquitinate the intracellular side of MHC I molecules and subsequently binds Ubc13 to Lys63-linked ubiquitinate the monoubiquitinated MHC I molecules [274, 275]. The ubiquitinated MHC I molecules are endocytosed, which requires the UIM-containing endocytosis adaptor epsin and clathrin [277]. The use of viral E3s to manipulate infected host cells through modulation of E2 enzymes is likely a widespread viral strategy. In addition to viral Tax-mediated E2/E3 manipulation and the employment of the viral K3 E3 to influence the host cell ubiquitination system, the poxvirus RING E3 p28 also hijacks multiple host cell E2 enzymes to enhance virulence [278, 279].

### Ubc13 in the response to bacterial infections

*Shigella flexneri* is a pathogenic bacterium that causes the human intestinal disease shigellosis by invasion of the intestinal epithelial cells [280]. *S. flexneri* encodes a glutamine deamidase protein, OspI, which is secreted from the bacteria to modulate host cell signaling [281]. OspI targets and deamidates host cellular Ubc13 at Gln100, which converts it to a glutamic acid residue and impairs its binding to TRAF6 to effectively inhibit NF-κB activation [281]. Multiple studies of OspI/Ubc13 complex crystal structures reveal the mode of OspI binding to Ubc13, which notably overlaps with known E3 and DUB binding surfaces [282, 283]. Interestingly, *Shigella flexneri* employs a similar strategy to further manipulate the host cell ability to fight the infection, through binding of its OspG effector kinase to the ubiquitin conjugated host E2 enzymes (E2~Ub), UbcH5 and UbcH7 [284, 285]. OspG also targets the NF-κB pathway, by preventing degradation of IκB (Figure 5).

The enteropathogenic *Escherichia coli* bacterium manipulates the host immune system through production of a methyltransferase protein, NleE [286]. Instead of targeting Ubc13 directly, NleE modifies zinc finger cysteines of TAB2/3 (Figure 5), which disrupts the critical Lys63-linked ubiquitin chain binding capacity of TAB2/3. NleE activity was shown to suppress NF-κB activation [286].

*Helicobacter pylori* is a bacterium that causes chronic inflammation in the gastrointestinal tract that can lead to the onset of gastric cancers and other ailments [287]. Upon infection, *H. pylori* activates the NF-κB pathway and causes an increase in host cell pro-inflammatory cytokines [288]. Lamb et al. demonstrated that an *H. pylori* protein, CagA, binds TAK1 and enhances its Lys63-linked ubiquitination, thereby activating the NF-κB pathway [289] in a Ubc13/Uev1A dependent manner [288].

### Ubc13 targeting in parasitic infections

Modulation of host immune responses by the ubiquitination system is not only limited to viral and bacterial infections, but also plays a role in how cells respond to parasitic infections. *Leishmania donovani* is a parasite that infects macrophages, which can cause them to undergo apoptosis [290]. *L. donovani* is responsible for visceral leishmaniasis (VL) disease, which causes enlargement of the spleen and liver, and can result in death. Gupta et al. demonstrate that *L. donovani* modulates host cell immune signaling by preventing protein complexes involved in TLR4-induced signaling from translocation from the membrane to the cytoplasm, affecting the levels of membrane-associated Ubc13 and decreasing Lys63-linked ubiquitination of TRAF6 [291]. Collectively the effects of *L. donovani* are thought to lower the production of proinflammatory cytokines likely through modulation of signaling pathways leading to NF-κB activation.

## UBC13 ROLE IN CANCER AND DEVELOPMENT OF ANTI-CANCER DRUG RESISTANCE

Two recent studies have strongly linked Ubc13 to regulation of breast cancer metastasis [155, 292]. As previously described above, TGF leads to p38 and JNK activation through a non-SMAD signaling cascade that depends on Ubc13/TRAF6 Lys63-linked auto-ubiquitination to activate TAK1 and the downstream MAP kinases. It was demonstrated that Ubc13 is upregulated in tumor tissue samples of the breast, prostate, colon, pancreas and in lymphoma [155, 293] and that upregulated Ubc13 in breast cancer is correlated with reduced survival. Xenograft studies of breast cancer in a mouse model system showed that Ubc13 expression was not strongly linked to primary tumor growth, however Ubc13 expression was required for the development of metastasis [155]. The authors also found that Ubc13 silencing in the LM2 human breast cancer cell line inhibited TGFβ-induced activation of p38, but not JNK activation. Rescue of the Ubc13-silencing effect by the expression of a constitutively active MKK downstream of Ubc13 in the pathway demonstrated correct pathway identification of the Ubc13-p38 signaling as responsible for the breast cancer cell metastasis [155]. Additionally, Uev1A overexpression in a different breast cancer cell line (MDA-MB-231) promoted metastasis in a Ubc13-dependent manner, which was attributed to activation of the NF-κB transcription factor [292]. Finally, further support for a role of Ubc13 in cancer metastasis has come from a study of Ubc13/Uev1A orthologs in *Drosophila* that demonstrated a regulatory role of Ubc13/Uev1A in JNK-dependent tumor metastasis and growth [294–296].

Cisplatin is a well-known, frequently used chemotherapeutic agent that primarily causes purine base-linked DNA inter- and intrastrand crosslinks. Cisplatin-mediated DNA crosslinks stall DNA replication, which leads to collapse of replication forks and subsequent generation of DNA DSBs [297]. Unfortunately, anti-cancer drugs such as cisplatin provide a selective pressure, leading to the evolution of resistant cancer cells. Su et al. found that chronic cisplatin treatment of nasopharyngeal carcinoma (NPC) cells results in upregulated expression of the TS, FA and HR DNA repair pathways. Cisplatin-resistant NPC cells (cr-NPCs) show increased Ubc13 expression, and have a higher frequency of sister chromatid exchange (SCE). Depletion of Ubc13, as well as other DNA repair genes, resensitized the cr-NPCs to cisplatin and suppressed SCE [297]. Similarly, a study in fission yeast found deletion mutants of Ubc13 render yeast hypersensitive to cisplatin [298].

cBioPortal is a database of sequenced cancer genomes, which maps mutations from a large pool of samples for a given protein onto its structure if one is available [299]. The functional impact of a given mutation in a cancer is largely based on residue conservation in protein families, and mutations are rated low, medium, or high, with medium/high ranked as possible driver mutations [300]. It is therefore likely that differences in populations and genetics may result in differences in the effects of Ubc13 mutations in cancers and cancer susceptibility, as for example, one study found a lack of breast cancer predisposition with mutations in Ubc13, Mms2, and RNF8 in a Northern Finnish population [301]. Ubc13 cancer mutations in positions likely to disrupt the active site structure are Thr73Ile (liver), Ile75Met (uterine), Pro78Leu (melanoma), and Arg85Ile/Lys (lung adenocarcinoma/multiple myeloma). Ubc13 mutations that could destabilize the loop that opposes the active site (114-124) are Ser113Thr (liver) and Ala126Val (colorectal). Active site structure-disrupting mutations and active site loop mutations would likely hinder proper transfer of the covalently linked donor ubiquitin from the E2 to an acceptor lysine on a substrate or another ubiquitin. This would likely lead to impairment of DDR factor recruitment to the sites of DNA DSBs and disruption of immune signaling pathways. It is possible then, that these particular cancers may have inefficient HR repair, which could possibly be exploited in treatment strategies, such as synthetic-lethality or sensitivity to PARP inhibitors. Additionally, the cancers with Ubc13 active site mutations may rely on non-Ubc13 dependent pro-survival pathways, which could be selectively targeted to kill the cancer cells. Ubc13 Arg70Leu/Cys (lung/stomach adenocarcinoma) mutations likely affect Mms2 binding, as Arg70 hydrogen bonds to the main chain carbonyl of Mms2 Met41. These mutations may cause Ubc13 to be less specific for creating Lys63-linked ubiquitin chains, and likely disrupt ubiquitin chain formation altogether [302]. The Ubc13 mutation Arg7Ser is found in liver hepatocellular carcinoma and small cell lung cancer. It is predicted to have a high functional impact due to its high degree of sequence conservation. Arg7 makes hydrogen bonding interactions that stabilize Ubc13 internal structure and may facilitate E3 interactions. An internal hydrogen bond is formed with the main chain carbonyl oxygen of Leu99, which is the first residue C-terminal to the conserved Ubc13 Ser-Pro-Ala motif important for RNF8 binding [38] and likely other E3s such as CHIP [32]. Ubc13 Arg7 also likely hydrogen bonds with the main chain carbonyl oxygen of RNF8 Ile405, which may be a major E2 selectivity determinant. The effects of the Ubc13 Arg7Ser mutation would likely destabilize E3 binding, which could cause major defects in DDR repair factor recruitment and possibly other pathways where Lys63-linked ubiquitin chains are necessary.

## UBC13 AS A TARGET FOR INHIBITOR DEVELOPMENT

There has been considerable interest in discovering and developing Ubc13 inhibitors, largely owing to the role of Ubc13 in various DDR and cytoplasmic signaling pathways linked to cancer. Several natural compounds isolated from marine sponge species with proposed activity against Ubc13 have been reported, including a β-carboline alkaloid isolated from *Luffariella variabilis* [303], manadosterols A/B from *Lissodendryx fibrosa* [304], and leucettamol A from *Leucetta aff. microrhaphis* [305]. Small-molecule inhibitors of Ubc13 have also been discovered including Ubc13/Uev1A protein-protein antagonists [306], and active site-targeting covalent inhibitors [27, 307–309]. The β-carboline alkaloid isolated from *Luffariella variabilis* was reported to inhibit Ubc13 interaction with Uev1A using an *in vitro* ELISA assay (IC_50_ of 20 μM), and also had inhibitory effects on the proteasome [303]. The manadosterol A/B inhibitors were also reported to inhibit Ubc13 interaction with Uev1A with IC_50_ values of 0.09 and 0.13 μM for manadosterol A and B, respectively [304]. The inhibition of Ubc13/Uev1A by manadosterol A/B compounds were also monitored using an *in vitro* ELISA assay. Leucettamol A from *Leucetta aff. microrhaphis* was the first reported marine sponge-derived inhibitor of Ubc13 interaction with Uev1A, with an IC_50_ of 106 μM as determined by an *in vitro* ELISA assay [304, 305].

A report in 2012 [310] employed time-resolved fluorescent resonance energy transfer (TR-FRET) to identify and characterize a small-molecule Ubc13 inhibitor, ML307. The report suggested that ML307 has a sub-micromolar affinity for Ubc13, with an IC_50_ of 781 nM. We, however, did not detect any inhibition of diubiquitin production by Ubc13/Mms2 in the concentration range of 2-80 μM, nor any inhibition of polyubiquitin chain production in the presence of a RING E3 ligase construct (RNF8_345-485_/Ubc13/Mms2) in the concentration range of 1-10 μM (unpublished data).

Scheper et al. used a library of N-alkylglycine derivatives termed peptoids to screen for Ubc13-Uev1A interaction inhibitors using a yeast-two hybrid assay, along with virtual screening. They identified two compounds they named compounds Ia and IIa, and with surface plasmon resonance-determined IC_50_ values of 10 pM and 1.1 μM, respectively [306]. Several *in vitro* assays suggested that both compounds inhibited Ubc13 binding to Uev1A, as well as ubiquitination activity. Additionally, several assays done using HeLa cells suggest compound Ia inhibits TNFα-induced NF-κB activation [306].

Pulvino et al. carried out a HTS screen for compounds that inhibit NF-κB-mediated gene expression and uncovered the compound NSC697923 as a Ubc13 small-molecule inhibitor [307]. The structurally related compound BAY 11-7082, previously thought to inhibit IKKs, was also recently identified as a relatively non-specific E2 inhibitor [308]. Our group [27] and Strickson et al. [308] confirmed both NSC697923 and BAY 11-7082 to be covalent inhibitors of Ubc13, and our lab solved co-crystal structures of both inhibitors bound to Ubc13 in 2015. Both NSC697923 and BAY 11-7082 inhibit NF-κB activation and the DDR [27, 307, 308]. Based on our structural data, we designed a mutant that changes the orientation of the Ubc13 active site loop, blocks the pocket that the inhibitor-adducts would occupy, and effectively resists NSC697923 *in vitro*, but maintains the ability to build ubiquitin chains. Ubc13-knockout MEFs reconstituted with the resistant mutant showed significant resistance to inhibition in DNA damage and NF-κB signaling by NSC697923 compared to a wild type control. This study demonstrates that compounds related to NSC697923 offer promise for the selective inhibition of Ubc13 over other possible E2 enzymes [27]. The BAY 11-7082 compound is a less specific inhibitor compared to NSC697923 and also inhibits protein tyrosine phosphatases [27, 311]. Interestingly, both inhibitors target several DUBs, particularly USP7 [312]. BAY 11-7082 has been shown to have anti-cancer effects on T-cell lymphomas [313], B-cell lymphomas [314], colon cancer cells [315] and HTLV-1 T-cell lines [316]. Cellular studies using the more recently discovered NSC697923 are still only preliminary, however anti-cancer properties against neuroblastoma [309] and diffuse large B-cell lymphoma cells have been reported [307].

## A NOVEL MECHANISM FOR ACTIVATION OF UBC13

**Figure 6:**
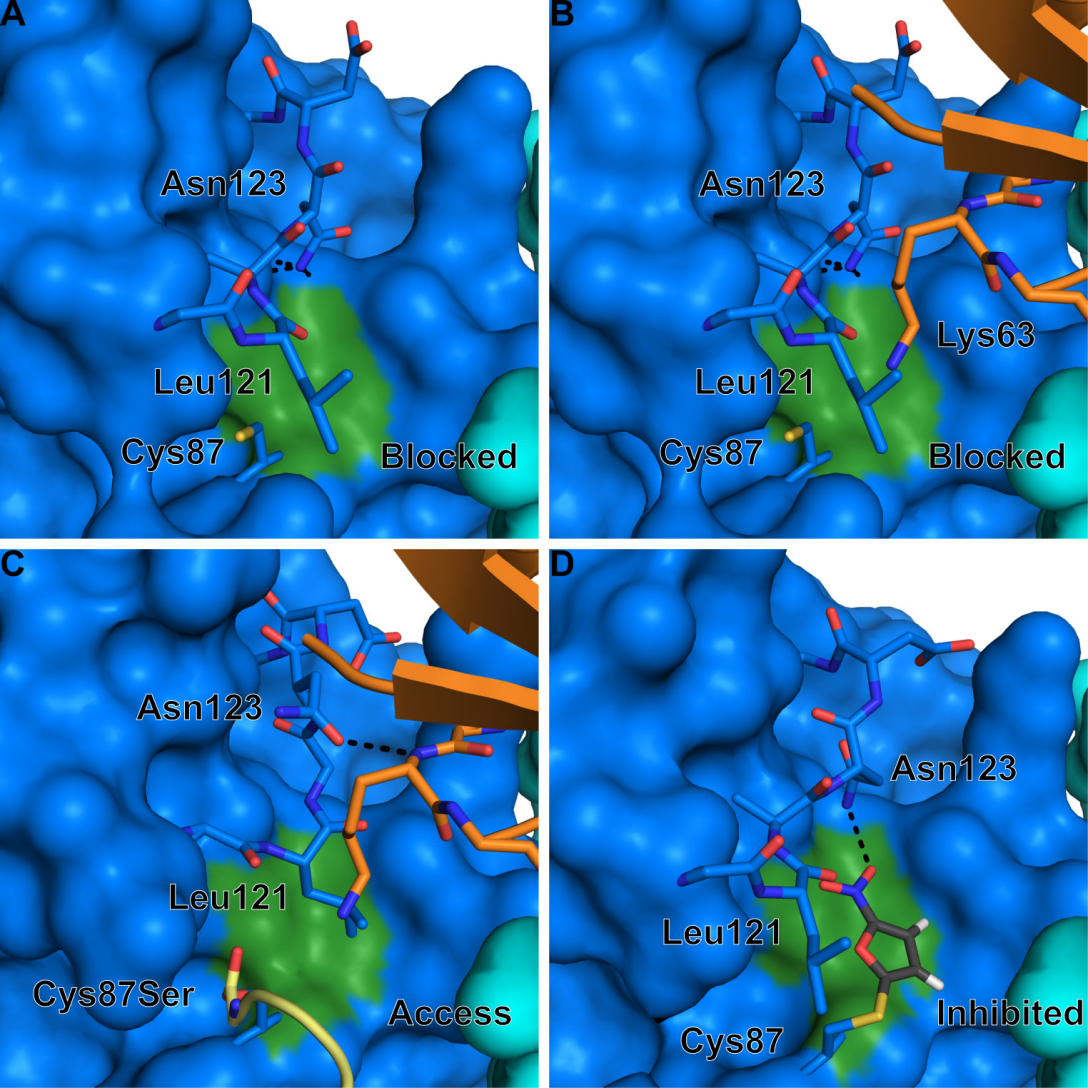
Ubc13 activation model requiring active site loop re-orientation **A.** Ubc13/Mms2 structure shows the inactive loop conformation where access to the active site cysteine (Cys87) is blocked by Ubc13 Leu121. The loop orientation is stabilized by Ubc13 Asn123, which hydrogen bonds with the main chain of His77, Pro78, and Val80. (PDB: 1J7D). **B.** Same structure as **A.** with the acceptor ubiquitin from 2GMI overlaid to highlight blockage of the approach of the Lys63 residue of a potential acceptor ubiquitin (PDB: 1J7D). **C.** Structure of yeast Ubc13~Ub/Mms2 with a symmetry related acceptor ubiquitin showing the shifted E2 active loop conformation of the Ubc13 active site loop to allow Lys63 access to the active site cysteine residue (PDB: 2GMI). Ubc13 Asn123 stabilizes the active loop conformation in Ubc13 by hydrogen bonding to the main chain of the incoming acceptor ubiquitin Lys63 residue. Ubc13 Leu121 is shifted into the small pocket in the active loop conformation. **D.** Structure of Ubc13/Mms2 after NSC697923 has reacted with the active site cysteine residue to leave behind a 5-nitrofuran adduct, which stabilizes Ubc13 active site loop in the inactive conformation (PDB: 4ONM). Ubc13 is blue, Mms2 is cyan, donor ubiquitin tail is yellow, acceptor ubiquitin is orange and reacted NSC697923 (5-nitrofuran adduct) is gray. The small Ubc13 pocket next to the active site cysteine is colored green. Surface representation is used for Ubc13 and Mms2. Stick representation is used for Ubc13 active site loop and cysteine, the acceptor ubiquitin Lys63, and the donor ubiquitin C-terminal tail. Labels correspond to human Ubc13 residues.

We suggest that Ubc13 initially exists in an inactive ground state governed by the conformation of the active site loop (residues 114-124). In structures of Ubc13 lacking ubiquitin or an E3 partner, the active site loop adopts a conformation that blocks access to the active site cysteine through the positioning of Leu121 (Figure 6A, 6B). This orientation of the active site loop is only observed in Ubc13 and not in any other E2 analyzed to date. The orientation is not merely a crystal packing artifact since it is conserved in the structure of the *S. cerevisiae* Ubc13 ortholog [23]. This inactive loop conformation shifts to allow the incoming Lys63 of an acceptor ubiquitin access to the active site cysteine for the nucleophilic attack. This is observed in the structure of Mms2/Ubc13~Ub (PDB: 2GMI [19]), in which a symmetry mate of the donor ubiquitin binds Mms2 in a neighboring asymmetric unit, providing a view of an acceptor ubiquitin with its Lys63 directed towards the Ubc13 active site (Figure 6C). This active conformation is observed in 17 of the other human E2s suggesting that this conformation is important for the catalytic activity of many E2s [27]. In Ubc13, part of the trigger involved in the transition from the inactive to active loop conformation likely involves Asn123, which is only found in Ubc13 and no other E2 family members. In the inactive loop conformation, Asn123 is buried within Ubc13 and hydrogen bonds to the main chain carbonyl oxygens of His77, Pro78, and Val80 (Figure 6A, 6B). In the active conformation, Asn123 is rotated out to hydrogen bond with the main chain amide of the incoming acceptor ubiquitin Lys63, catalyzing the reorientation of the entire loop (Figure 6C). Based on NMR measurements and molecular dynamics simulations, we have suggested that the Ubc13 active site loop is dynamic and that changes in the loop conformation could serve as a catalytic gate [57]. Intriguingly, the structures of Ubc13 bound to a variety of E3s or other regulatory factors such as OTUB1 and OspI reveal a variety of active site loop conformations, suggesting that the binding of these factors could influence Ubc13 catalytic activity through effects on the active site loop.

Insights from the Ubc13 loop orientation observations suggest a way forward for rational Ubc13 inhibitor development. In the NSC697923-inhibited Ubc13 structure (Figure 6D), Asn123 hydrogen bonds to the 5-nitrofuran adduct, which packs into the pocket formed by the active site loop and effectively locks the loop in its inactive conformation. We suggest that covalent attachment may not be necessary to selectively inhibit Ubc13, as a compound that could non-covalently bind the small Ubc13 pocket, while forming a hydrogen bond to Asn123, might still trap the loop in its inactive conformation to block Ubc13 catalytic activity.

## CONCLUSIONS AND PERSPECTIVE

In this review, we have attempted a global review of studies of Ubc13 in various pathways and capacities. We focused on studies that directly implicate Ubc13, however we note that we have omitted studies that have implicated a role for Lys63-linked ubiquitin chains in particular cellular processes, but have not explicitly demonstrated a role for Ubc13. As the sole known E2 capable of generating Lys63-linked ubiquitin chains, Ubc13 represents a critical protein in many pathways in many different cell types. The fact that Ubc13 is crucial for inflammatory and DNA damage response pathways involved in many different cancer types, makes it an attractive target for therapeutic modulation. Additionally, Ubc13 involvement in viral, bacterial, and parasitic infections makes it an even more important enzyme in human disease. Ubc13 possesses a number of desirable traits as a target for inhibition. Its unique active site architecture within the E2 family presents possibilities for the development of Ubc13-specific inhibitors [27]. In addition, a subset (17 E2s) of the ~34-40 active human E2 enzymes share a great degree of similarity in overall fold and residue conservation, therefore the discovery of a unique active site loop conformation and small pocket crucial to Ubc13 inhibition sets it apart from other E2s.

To date no synthetic lethal relationships have been uncovered between Ubc13 and other genes in humans. The principle of synthetic lethality is powerful, and has been demonstrated through the application of poly (ADP-ribose) polymerase (PARP) inhibition as a means to treat BRCA-deficient cancers [194, 317]. The inhibition of PARP, involved in single-strand break repair, results in DNA lesions that require HR for repair and results in specificity for BRCA-deficient cancer cells over normal cells. This is particularly important because traditional chemotherapy targets highly proliferating (or dividing) cells, which causes severe side-effects such as a dangerously compromised immune system through bone marrow destruction [318]. The other issue with the traditional mode of targeting is that solid tumors are thought to have a gradient of proliferation rates with stagnant non-dividing cells at the core, and proliferating cells on the outer surface. This is likely a result of a unique tumor microenvironment characterized by hypoxia, and decreased diffusion rates of nutrients and acidity [318]. Indeed, many modern therapies that target DNA damage response proteins are currently being developed due to the involvement of DDR factors and genome instability in many cancers [319].

A potential Ubc13-dependent synthetic-lethal relationship has been identified in yeast, which may be present, but unidentified in humans. Rev3 is the catalytic subunit of DNA polymerase zeta, which functions in the error-prone TLS pathway (Figure 4B) [320, 321]. As previously discussed, Ubc13/Mms2 is important for the error-free lesion bypass TS pathway. Broomfield et al. [320] found that cells with mutated Mms2 (defective TS pathway) rely on the error-prone TLS, which requires Rev3 and that a double Mms2, Rev3 mutant was exceptionally sensitive to DNA damage induced by UV irradiation and alkylating methyl methanesulfonate (MMS) treatment. It is likely that Ubc13 shares the same relationship to Rev3 as Mms2, therefore the relationship could likely be exploited in humans if cancers are identified with mutant/defective Rev3. The Rev3-Mms2 relationship was not, however, observed in DT40 chicken cells [322], so the putative relationship between Rev3 and Ubc13/Mms2 in humans needs to be established.

We recently proposed an alternative method of modulating the ubiquitin system in relation to Ubc13 [45]. In this method, E2/E3 pairs are targeted as a single functional unit with inhibitor-protein interactions spread across the E2 active site and ubiquitin binding surface. Targeting strategies for the E2/E3 could start with covalent inhibitors anchored to the E2 active site cysteine. These starting compounds could be varied in HTS or rational screening approaches to maximize interactions with the nearby ubiquitin tail binding groove, as well as the E2/E3 surface. Such an approach could be used to develop compounds that inhibit specific E2/E3 pairs without the need for covalent reaction at the active site cysteine. Compounds that are highly selective for a particular E2/E3 pair could provide intriguing tools for the selective modulation of specific signaling pathways. It is likely that future studies will uncover still further Ubc13-dependent signaling roles, as its Lys63-linked ubiquitin chains are pervasive and seem to act as protein aggregation signals at important sites that require the assembly of large protein signaling complexes.
